# Improving the accuracy of distributed cooperative positioning at urban intersections based on mathematical analysis and robust filtering

**DOI:** 10.1371/journal.pone.0358244

**Published:** 2026-09-24

**Authors:** Dan Liu, Zhen Zeng, Ting Li

**Affiliations:** 1 Personnel Department, Heilongjiang Open University, Harbin, China; 2 Teaching Department, Heilongjiang Open University, Harbin, China; Beijing Institute of Technology, CHINA

## Abstract

To improve the cooperative positioning accuracy of urban intersections under conditions of global positioning system signal blockage and complex traffic flow, this paper proposes a cooperative positioning model that integrates a mathematical analytical Gaussian belief propagation robust filtering algorithm with a spatial attention mechanism. A hardware verification platform integrating roadside sensing units, connected test vehicles, and 5G-vehicle-to-everything communication is constructed to achieve distributed cooperative positioning analysis of urban intersections. The results show that the proposed fusion model exhibits the best overall performance in intersection cooperative positioning, with a root mean square error of only 0.15 m, significantly lower than both single algorithms and the spatiotemporal attention model. The geometric accuracy attenuation factor is as low as 1.8, the effective cooperative gain reaches 60%, and the positioning coverage rate reaches 98%. However, experimental results under ring-topology, high-density congestion, and communication failure scenarios indicate a noticeable performance degradation, suggesting that the robustness of the proposed method is limited under extreme network conditions. The integrated design of “mathematical model analysis - dynamic feature extraction - vehicle-road cooperative fusion” can effectively achieve a leap in intersection positioning accuracy from single-vehicle intelligence to group collaboration, providing key technical support for reliable perception of smart intersections and connected autonomous driving.

## 1. Introduction

As a key node in the transportation network, the high-precision and high-reliability collaborative positioning of urban intersections is the core technical foundation for supporting the safe passage and efficient management of intelligent connected vehicles, and directly affects the overall efficiency and safety of urban intelligent transportation systems [1]. With the development of “vehicle-road-cloud integration” technology and autonomous driving, urban intersection environments have become typical scenarios where Global Navigation Satellite System (GNSS) signals continue to deteriorate due to factors such as building obstruction, dynamic traffic flow interference and multipath effects, posing a severe challenge to existing positioning technologies [2]. Traditional positioning methods mostly rely on single sensors or simple data fusion, which makes it difficult to maintain stable accuracy in complex obstruction environments, especially lacking the ability to deeply mine and dynamically optimize the collaborative perception information between vehicles and between vehicles and roads [3]. Distributed collaborative positioning refers to the construction of a dynamic network by multiple moving or stationary intelligent agents through information interaction and collaborative computing in an environment without absolute reference benchmarks or with limited reference signals. Therefore, promoting the evolution of urban intersection positioning technology towards “distributed collaboration” and “intelligent integration” has become an urgent need for industry development. Cooperative positioning aims to overcome the limitations of single-vehicle perception and improve the accuracy of global positioning by sharing and jointly solving multi-source information between vehicles and roads [4].

In their research, Jin et al. aimed to understand the role of the GNSS. The latest progress in cooperative positioning of GNSS is presented in a comprehensive survey, and the latest work is analyzed and compared in six parts, including architecture. The results showed that GNSS faced challenges in urban vehicle networks, and GNSS-based cooperative positioning faced challenges in practical and academic applications. Potential research directions were also outlined [5]. To improve the positioning and tracking performance of dynamic user equipment in broadband millimeter wave systems, Guo et al. proposed a ping-pong positioning framework driven by the lower bound of positioning error. Multi-dimensional information fusion was used to assist positioning, realize the evaluation of the positioning error limit by the mean square composite position error boundary, optimize the hybrid beamformer and multi-path cooperative positioning by the alternating optimization algorithm. The results showed that compared with the scheme of unoptimized beam configuration, the method improved the estimation accuracy by at least 16% and only required about a quarter of the time slot resources [6]. To optimize the collaborative performance of connected autonomous vehicles at unsignalized intersections and explore its practicality, Wang et al. proposed a deep reinforcement learning method based on the shared advantage A2C model and asynchronous training strategy. The results showed that the method improved the retreat time and average latency by 30% and 40% respectively in the simulation, and the experiment verified its great application potential in real-world scenarios [7]. To solve the problem of unclear task division and communication objectives in the field of vehicle road collaborative perception, and to achieve rapid localization of specific research directions, Gao, X and others systematically analyzed the timing and role of each task in the collaborative perception workflow. The perception tasks were divided into three categories: detection, segmentation, and tracking, and the communication tasks were sorted out from the dimensions of delay robustness and bandwidth efficiency. The research results indicated that this classification framework could effectively guide the design of collaborative perception systems [1]. To address the problem of simultaneous localization and external calibration in multi-robot setups, Murai et al. proposed a novel, scalable, fully distributed, and online method. The results showed that this method could not only achieve accurate robot localization and automatic calibration, but also perform effectively under challenging conditions such as high-noise measurement, severe communication failures, or limited communication range [8].

Qian X et al. proposed a semi-supervised deep learning algorithm for direction of arrival estimation. This model enhanced unlabeled data by generating pseudo-labels. The results showed that this method was superior to supervised learning and other semi-supervised learning algorithms [9]. Zhao Y et al. proposed a cloud based privacy protection distributed collaboration scheme to achieve efficient collaborative traffic at signalless intersections while ensuring the privacy and data security of autonomous driving vehicles in the Internet of Vehicles. The research results showed that this scheme encrypted vehicle state data through affine masking technology, and after solving the equivalent optimization problem in the cloud, the vehicle can recover the real controlled instructions through inverse masking, which can not only avoid collisions and protect data privacy, but also improve traffic efficiency and fuel economy, while reducing computational burden [10]. To solve the congestion problem at complex intersections in intelligent transportation systems and achieve efficient collaborative optimization of urban road traffic flow, Li Y et al. propose a vehicle path planning and prediction algorithm based on attention mechanism. The research results showed that the algorithm balanced the traffic flow at each intersection through attention mechanism, captured spatiotemporal correlations with CNN-LSTM architecture, and analyzed local spatial correlations, significantly shortening the average travel time and improving intersection throughput [3]. To improve traffic efficiency at urban intersection clusters and reduce delays, Ma C provided a systematic review of recent advances in signal coordination control and established a theoretical framework. The results indicated that research focused on dynamic multi-criteria classification, multi-objective optimization for signal coordination, the widespread application of deep reinforcement learning, and collaborative optimization [11]. Significant progress has been made in large-scale perception and collaborative decision-making in the field of vehicle positioning and connected vehicles (V2X). In the direction of roadside perception, Liu Y et al. proposed an information fusion method based on multi node roadside sensor collaboration, which achieves real-time detection and continuous tracking of multi-target vehicles in complex traffic scenes by constructing a distributed perception network. This type of method utilizes roadside infrastructure to continuously observe vehicle status, effectively improving target coverage and tracking stability in urban road environments. However, it still relies heavily on centralized processing structures for cross domain information fusion and vehicle status feedback [12]. In the direction of vehicle to vehicle collaborative communication, Zhang J Researchers have studied a formation control method that utilizes optical communication to achieve high-precision and low latency information exchange between vehicles. By enhancing queue coordination consistency through direct communication between workshops, the formation stability and response capability of vehicles in high-speed moving scenarios have been improved. However, this type of method mainly focuses on optimizing the communication link layer and control layer, and there is still relatively little consideration for unified probability modeling and uncertainty fusion of multi-source heterogeneous positioning information [13]. Liu et al. proposed a real-time vehicle tracking framework for large-scale roadside sensor networks (A framework for real-time vehicle tracking in large-scale roadside sensor networks). This method is based on multi access edge computing (MEC) architecture to fuse multi-source sensor data such as cameras and radars, and realize real-time tracking of vehicle tracks in complex road environments. The experimental results show that the framework can achieve continuous tracking of multiple vehicles in large-scale road networks while maintaining low positioning errors and computational delays. However, this method mainly relies on roadside sensor data fusion, and there are still deficiencies in the uncertainty modeling of different sensor sources in dynamic environment and the adaptive optimization ability of location information [14].

At present, various deep learning models have been used to achieve neighborhood localization analysis in different localization tests, but there is relatively little research on collaborative localization analysis of urban intersections. Therefore, how to achieve collaborative positioning and improve positioning accuracy at urban intersections remains a key research focus. Based on this, a new method is proposed to improve the accuracy of distributed collaborative positioning at urban intersections by combining the mathematical analysis Gaussian belief propagation (GaBP) robust filtering algorithm with spatial attention mechanism.

The innovations of this study are as follows: (1) Theoretical Innovation: The innovation of this study lies primarily in the collaborative modeling of traditional Gaussian belief propagation and spatio-temporal attention mechanisms within a unified factor graph framework, and in achieving bidirectional adaptive updating between observed and prior information by introducing an uncertainty-based weight modulation strategy. This method differs from existing collaborative localization methods based on fixed weights or unidirectional fusion, enhancing the flexibility and robustness of information fusion in complex dynamic environments.

(2) Technical Innovation: A distributed collaborative computing framework with low communication overhead is constructed, integrating the GaBP robust filtering algorithm with a spatiotemporal attention-based trajectory prediction network within a single factor graph optimization framework. A hardware verification platform is designed that integrates roadside sensing units, connected test vehicles, and 5G-V2X communication. A complete software workflow is developed, ranging from binary data decoding to standardized output, enabling real-time acquisition, collaborative processing, and closed-loop verification of multi-source heterogeneous sensors.

(3) Application Innovation: Addressing the practical challenges of severe GNSS signal obstruction and complex, dynamic traffic flows at urban intersections, this work provides a distributed collaborative positioning solution characterized by high accuracy, high robustness, and high coverage. Field test results demonstrated that the proposed fusion model achieved a positioning RMSE of only 0.15 m, an effective collaborative gain of up to 60%, and a positioning coverage rate of 98%, with a geometric accuracy degradation factor as low as 1.8. This successfully bridges the gap from single-vehicle intelligent perception to vehicle-road collaborative swarm intelligence, providing critical technical support for reliable perception at smart intersections and connected autonomous driving.

## 2. Research design

### 2.1. Collaborative localization of urban intersections based on mathematical analysis and robust filtering

Urban intersections are signal transmission base stations with poor GNSS signal and complex traffic flow. A dense distributed network formed by connected intelligent vehicles and roadside equipment enables V2V/V2I information coordination in specific urban areas. However, high-precision navigation equipment often incurs significant costs in these urban network areas. Therefore, how to achieve distributed navigation at intersections and improve collaborative positioning accuracy is a current research focus. To address this, GaBP is proposed, which can improve the information navigation and positioning accuracy of intersections by reducing communication load [15]. Robust filtering is not an independent algorithm but a design principle to improve the overall performance of the model. The GaBP algorithm uses a Markov random field as the information transmission model. During information transmission, the model first defines information transmission nodes and calculates the confidence of the nodes as shown in Equation (1).


$$\begin{array}{c} h\left(x_{i}\right)=\frac{\text{1}}{c_{i}}\varphi_{i}\left(x_{i,}y_{i}\right)\prod_{x_{j}\in R\left(x_{i}\right)}m_{x_{j}\to x_{i}} \end{array}$$ (1)

In Equation (1), $h\left(x_{i}\right)$ represents the confidence level of the node $x_{i}$, $c_{i}$ represents the normalization constant, $\varphi_{i}$ represents the node potential function, $y_{i}$ represents the node observation evidence network, $m_{x_{j}\to x_{i}}$ represents the set of all neighboring nodes $x_{j}$ of the node $x_{i}$, and ∏ represents the product. The changes before and after the information transmission process are shown in Equation (2) [16,17].


$$\begin{array}{c} m_{x_{j}\to x_{i}}=\sum_{x_{j}}\varphi_{j}\left(x_{j},y_{j}\right)\varepsilon_{ji}\left(x_{j},x_{i}\right)\prod_{k\in R\left(x_{j}\right)/\left\{x_{i}\right\}}m_{x_{k}\to x_{j}} \end{array}$$ (2)

In Equation (2), $\varepsilon_{ji}\left(x_{j},x_{i}\right)$ represents the edge potential function connecting the node $x_{j}$ and the node $y_{j}$, $R\left(x_{j}\right)/\left\{x_{i}\right\}$ represents the set of neighboring nodes of node $x_{j}$, excluding the message receiving node $x_{i}$. $m_{x_{k}\to x_{j}}$ represents the message passed from node $x_{j}$ ‘s neighbor k to $x_{j}$. The node potential function represents the prior state information obtained by a single vehicle node based on its own sensors, and quantifies the degree of matching between the current state estimation and the prior information. The edge potential function represents the relative constraints between adjacent vehicle nodes, such as the distance observations provided by ultra wideband ranging. The edge potential function quantifies the consistency between the actual ranging values and the predicted distance based on the two vehicle states. The confidence scores above are initialized to obtain the result shown in Equation (3) [18,19].


$$\begin{array}{c} h\left(x_{i}\right)=\frac{\text{1}}{c_{i}}\varphi_{i}\left(x_{i,}y_{i}\right) \end{array}$$ (3)

**Fig 1:**
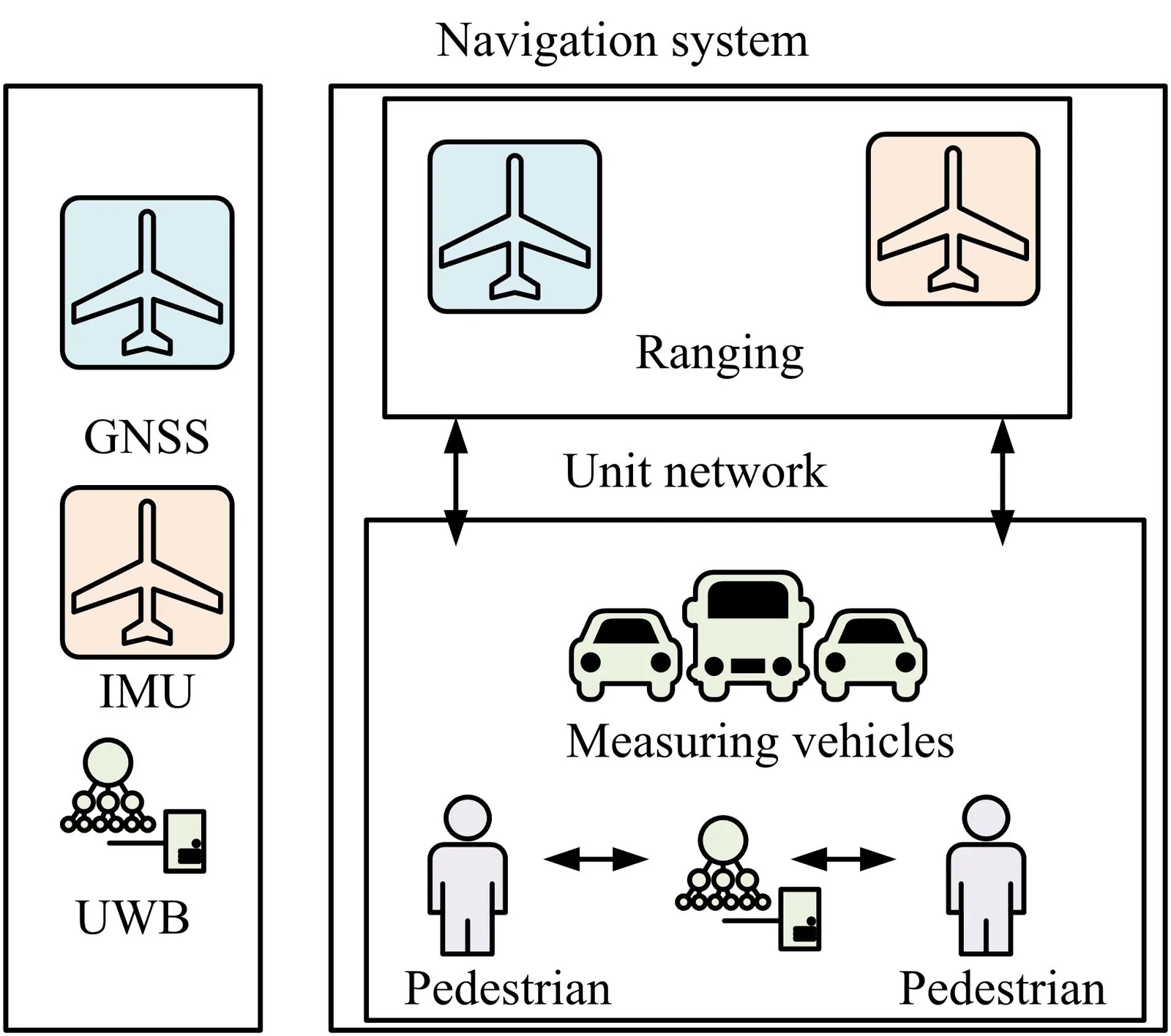
Collaborative navigation network.

Simultaneously, the confidence level is updated and judged. If the current confidence level has not reached the convergence state, the information transmission of Equation (2) is performed. If the confidence level has reached the convergence state, the minimum mean square error estimation analysis is performed to obtain the marginal probability change of the hidden node. The study uses the Markov random field model for information transmission. However, due to the large number of communication systems between vehicles and intersections in the cooperative navigation network, the navigation and positioning accuracy of the vehicle deteriorates. Therefore, the study introduces a navigation network to improve the positioning and navigation accuracy of the vehicle. Fig 1 shows the cooperative navigation network.

Fig 1 shows the navigation network structure. In the navigation network, intelligent connected vehicles equipped with high-precision GNSS/Inertial Measurement Unit (GNSS/IMU) and Ultra-Wideband (UWB) serve as positioning reference nodes. Meanwhile, vehicles and roadside equipment equipped with low-cost GNSS/IMU and UWB measure distances with the reference nodes through UWB and combine their own sensor information. Pedestrians or non-motorized vehicles in the network can access the network through mobile terminals carrying UWB. Each node measures distance to each other through UWB and combines GNSS and IMU data to achieve stable and high-precision collaborative positioning between vehicles, roadside facilities and pedestrians in complex environments such as intersections where signals are blocked. The positioning formula of the node is shown in Equation (4) [20,21].


$$\begin{array}{c} g_{k}^{T}=p(x_{k}^{t}|x_{k}^{t-1},z_{k}^{t,imu}) \end{array}$$ (4)

In Equation (4), $g_{k}^{T}$ represents an IMU factor connecting adjacent time states in the factor graph, p(•|•) represents the conditional probability distribution, $x_{k}^{t}$ represents the state vector of the unmanned system k at the current time t, $x_{k}^{t-1}$ represents the state vector of the unmanned system k at the current time t−1, and $z_{k}^{t,imu}$ represents the raw data obtained from the time content IMU measurement from time $z_{k}^{t,imu}$ to time $z_{k}^{t,imu}$. The GNSS node positioning results are shown in Equation (5).


$$\begin{array}{c} w_{k}^{T}=p(z_{k}^{t,gnss}|x_{k}^{t}) \end{array}$$ (5)

In Equation (5), $w_{k}^{T}$ represents a GNSS factor related to GNSS measurements in the factor diagram, and $z_{k}^{t,gnss}$ represents the GNSS observation value actually received and processed by the unmanned system k at time t. The UWB node calculation formula is shown in Equation (6) [22,23].


$$\begin{array}{c} s_{k,j}^{t}=p(q_{k,j}^{t,iwb}|x_{k}^{t},x_{l}^{t}) \end{array}$$ (6)

In Equation (6), $s_{k,j}^{t}$ represents the UWB ranging factor, and $q_{k,j}^{t,iwb}$ represents the distance between the systems k and l at time t t, actually measured using UWB technology. $x_{k}^{t}$ and $x_{l}^{t}$ represent the estimated state vector values of system k and system l, respectively. Assuming that the attention weight between two cars is A, the noise variance formula of the UWB ranging observation model is shown in Equation (7).


$$\begin{array}{c} \sigma_{i,j}^{\text{2}}=\sigma_{\text{0}}^{\text{2}}•exp(-\beta •\alpha_{i,j}) \end{array}$$ (7)

In Equation (7), $\sigma_{\text{0}}^{\text{2}}$ represents the standard noise variance, β represents the sensitivity coefficient, and $\alpha_{i,j}$ represents high attention. At this point, the node information matrix for constructing the factor is shown in Equation (8).


$$\begin{array}{c} \Delta_{i,j}=\frac{\text{1}}{\sigma_{i,j}^{\text{2}}}J_{i,j}^{T}J_{i,j} \end{array}$$ (8)

In Equation (8), $J_{i,j}$ represents the Jacobian matrix of the observable residual state. The variance formula after modulation is shown in Equation (9).


$$\begin{array}{c} \Delta_{i,j}=\frac{\text{1}}{\sigma_{\text{0}}^{\text{2}}•exp(-\beta •\alpha_{i,j})}J_{i,j}^{T}J_{i,j} \end{array}$$ (9)

**Fig 2:**
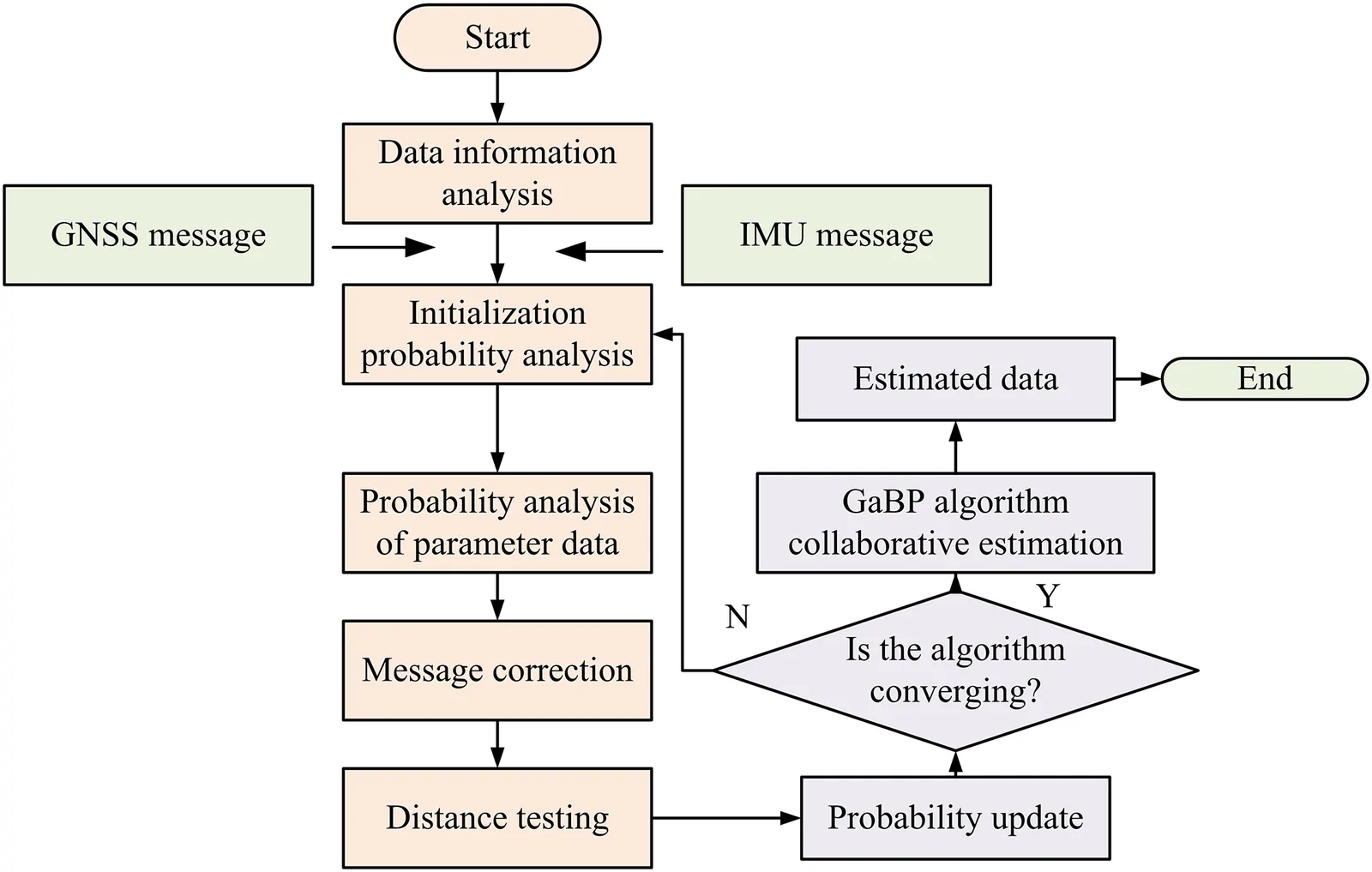
GaBP algorithm’s navigation system operation process.

The larger the attention weight in Equation (9), the larger the information matrix, and the higher the accuracy weight assigned to the relative observation between high crossing vehicles in information transmission. The navigation system performs cluster navigation analysis on vehicles equipped with sensors at urban intersections. Fig 2 shows the navigation system operation process using the GaBP algorithm.

As shown in Fig 2, during the navigation process, the algorithm first analyzes the observed data and then performs initial probability analysis. This probability analysis involves obtaining and analyzing the probability of parameter data from the navigation system. Next, message correction and distance testing are performed using data from the autonomous vehicle and the car. The overall data probability is then updated, and the algorithm is judged to have converged. If converged, collaborative estimation is performed using the GaBP algorithm. If converged, the state vector is updated, and the corrected message is recalculated. The process ends after successful collaborative estimation, outputting the system’s navigation solution. During the integration of the GaBP and spatial attention modules, the high-precision state from the GaBP forward pass provides accurate initial values for attention-based prediction. In turn, the intention probability and predicted trajectory from the attention module’s backward feedback are converted into dynamic motion constraint factors. These factors then adjust both the IMU noise covariance in the GaBP factor graph and the information weight of the UWB ranging factors. Meanwhile, when the positioning result is significantly different from the predicted trajectory, the confidence of abnormal observations is automatically reduced, thus achieving a closed loop of mutual correction between positioning and prediction.

### 2.2. Cooperative lane avoidance and trajectory localization prediction at urban intersections

**Fig 3:**
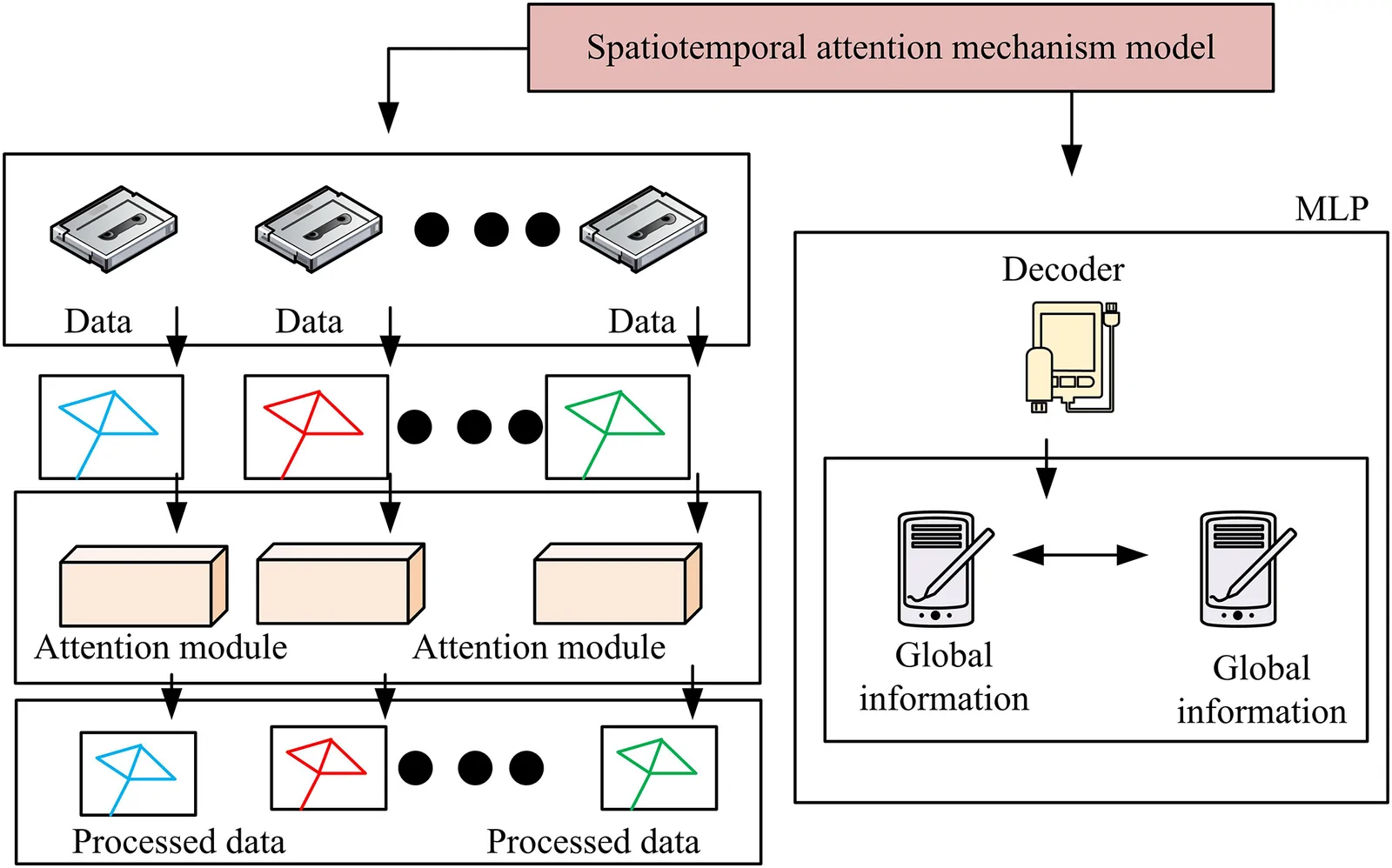
Structure of spatiotemporal attention trajectory prediction network.

The collaborative positioning method based on GaBP has achieved accurate estimation of vehicle status, but relying solely on historical observations to filter updates is difficult to cope with dynamic and complex intersection scenes. The future trends in sports, driving intentions, and interactions with other traffic participants are equally crucial for maintaining continuous positioning accuracy. Therefore, while achieving collaborative positioning at urban intersections, it is also necessary to reduce the probability of traffic accidents at urban intersections by analyzing vehicle driving intentions. Fig 3 shows the structure of the spatiotemporal attention trajectory prediction network.

**Fig 4:**
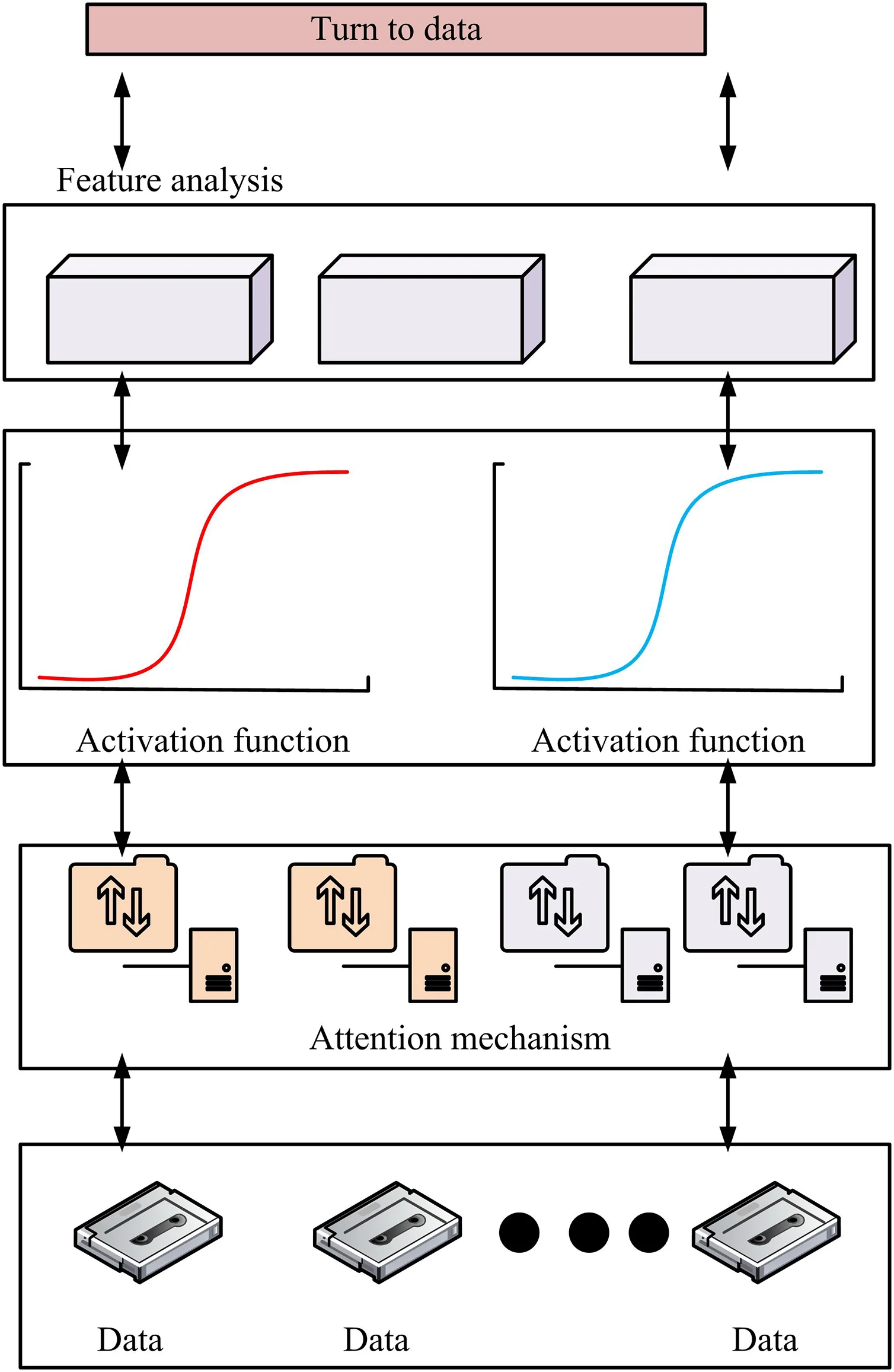
Spatiotemporal attention mechanism model.

As shown in Fig 3, the overall operation of the prediction network consists of two parts. The first part takes 20 consecutive frames of historical trajectory data as input. Each frame first passes through a spatial attention module to extract interaction features between traffic participants; the processed sequence features then pass through a temporal attention module to capture dynamic temporal dependencies. The second part of the model uses a Multi-Layer Perceptron (MLP). The model combines driver intent information with vehicle-road attention mechanism to achieve global environmental information interaction. The outputs of the two models and global information are input into the multimodal MLP decoder to generate multiple possible predicted trajectories, achieving end-to-end trajectory prediction that integrates driving intent, spatial attention, and global interaction. Vehicle intent, as the main factor affecting vehicle trajectory, determines the future trajectory changes and prediction accuracy of the vehicle [24,25]. The spatial attention mechanism model is used to solve how to accurately quantify and extract the spatial influence relationships between multiple traffic participants in complex intersection scenarios. Therefore, in order to more accurately predict vehicle operation, the spatial attention mechanism is used to analyze the driving intent of vehicles. Fig 4 shows the spatial attention mechanism model.

As shown in Fig 4, the network model first outputs the vehicle’s trajectory intention data through feature analysis, then processes the driving intention data of each frame using a spatial attention mechanism, then performs feature analysis on the processed data through an MLP attention model, and finally outputs the data information through an activation function to obtain the vehicle’s steering information data. The vehicle steering intention probability is shown in Equation (10) [26–28].


$$\begin{array}{c} p_{i}(y=j)=\frac{e^{o_{i}\left(j\right)}}{\sum_{k=0}^{\text{2}}e^{o_{i}\left(k\right)}} \end{array}$$ (10)

In Equation (10), $p_{i}(y=j)$ represents the probability that the i th vehicle belongs to the j th type of driving intention, $o_{i}\left(j\right)$ represents the j th element in the vector $o_{i}$, k represents the summation index, i represents the i th vehicle, and j represents the driving intention category. When j=0, it indicates the current vehicle is in a straight-going state; when j=1, it indicates the current vehicle is in a left-turning state; and when j=2, it indicates the current vehicle is in a right-turning state. The data parameters are trained using the cross-entropy loss function to obtain the values shown in Equation (11) [29–31].


$$\begin{array}{c} U_{i}=-\sum_{j=1}^{\text{3}}\left[q_{i}\left(y=j\right),log\left(p_{i}(y=j)\right)\right] \end{array}$$ (11)

In Equation (11), $U_{i}$ represents the cross-entropy function loss value of the i th vehicle. Simultaneously, the model performs trajectory prediction based on vehicle operation information through information fusion, and inputs the fused data as global feature data into the decoder. GaBP is essentially equivalent to iteratively solving the following quadratic objective function under a Gaussian diagram model.


$$\begin{array}{c} \underset{x}{min}\frac{\text{1}}{\text{2}}x^{T}Jx-h^{T}x \end{array}$$ (12)

Where J is the information matrix. In this method, spatial attention weights are introduced to modulate the observed covariance.


$$\begin{array}{c} R^{;}=\alpha R \end{array}$$ (13)

α represents spatial attention weight. The information matrix modulated with attention is denoted as.


$$\begin{array}{c} J^{,}=J_{GaBP}+△J\left(\alpha \right) \end{array}$$ (14)

Where △J(α) is a bounded perturbation term. The overall iterative update can be regarded as a bounded perturbation system based on the contraction mapping.


$$\begin{array}{c} x^{t+1}=F\left(x^{t}\right)+\vartheta \left(\alpha \right) \end{array}$$ (15)

When $F\left(x^{t}\right)$ satisfies the contraction condition and the perturbation term ϑ(α) is bounded, according to Banach fixed point theory, the system iteration still converges to a stable solution in the neighborhood without divergence. When the information matrix satisfies the strict diagonal dominance condition, the GaBP algorithm can ensure convergence. Establish an overall information matrix


$$\begin{array}{c} △=D+R \end{array}$$ (16)

D represents the diagonal matrix, and R represents the diagonal correlation matrix. The matrix satisfies the Walk Summability condition. Further define the GaBP iteration matrix


$$\begin{array}{c} T=-D^{-1}R \end{array}$$ (17)

The convergence condition is:


$$\begin{array}{c} \rho \left(T\right)<1 \end{array}$$ (18)

ρ(T) represents the spectral radius of the matrix. After introducing spatial attention modulation.


$$\begin{array}{c} \beta <\frac{\text{1}}{\|A\|} \end{array}$$ (19)

According to Banach fixed point theorem, the iterative process.


$$\begin{array}{c} J^{\left(k+1\right)}=F\left(J^{\left(k\right)}\right) \end{array}$$ (20)

There exists a unique fixed point.


$$\begin{array}{c} J^{\left(*\right)}=F\left(J^{\left(*\right)}\right) \end{array}$$ (21)

The GaBP model with added spatial attention still maintains convergence stability and will not cause information propagation divergence due to dynamic weight adjustment. Previous studies have shown that GaBP can achieve stable estimation performance when satisfying diagonal dominance and spectral radius constraints. For example, Murai et al. used Gaussian belief propagation to achieve distributed synchronous positioning, which maintains stable convergence performance even in the presence of communication noise [32].

**Fig 5:**
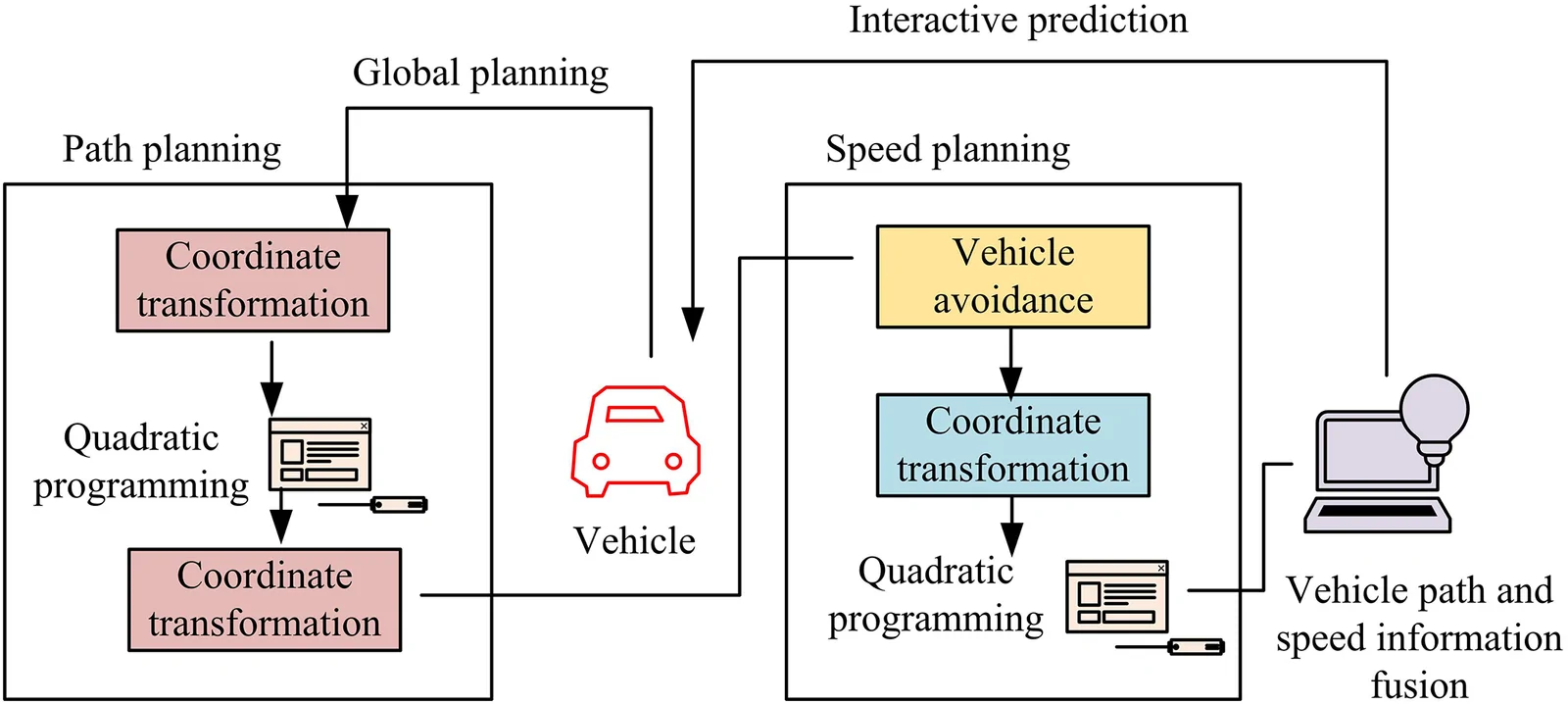
Structure of vehicle trajectory planning framework built for research.

After prediction planning, in order to achieve vehicle trajectory planning at urban intersections, the study will also combine the global vehicle path with the vehicle prediction results to perform intersection optimization analysis. More information about vehicle operating speeds and trajectories will be planned. Fig 5 shows the vehicle trajectory planning framework structure built in this study.

As shown in Fig 5, the trajectory planning framework during operation planning mainly consists of two parts: path planning and speed planning. Path planning involves transforming the vehicle’s position coordinates at the intersection for global planning, then transforming the coordinates again for secondary path planning, and finally projecting the vehicle’s trajectory. Simultaneously, speed planning is performed for vehicle trajectory avoidance decisions, resulting in secondary speed planning. Furthermore, by fusing vehicle path and speed information, more intersection interaction prediction information is obtained from the V2X network, and local path planning is then performed based on the interaction state information. The collaborative localization system passes the state estimation results to the trajectory prediction network. The driving intent and prediction confidence generated by the trajectory prediction network are then fed back to the localization module to dynamically adjust the range measurement confidence weights and the strength of motion constraints. This enables localization and prediction to mutually reinforce each other within a closed-loop “estimation–prediction–correction” cycle, achieving integrated coupling between information fusion and trajectory planning.

The bidirectional coupling framework of Gaussian belief propagation (GaBP) and spatial attention mechanism proposed in the study is essentially an adaptive information adjustment mechanism based on a structured probability graph model. This method is not strictly a globally optimal analytical solution, but rather achieves dynamic recalibration of information weights in the factor graph by introducing attention weights into the observation covariance adjustment term. At the same time, the driving intention output by the trajectory prediction module is fed back to the IMU process noise update term as a prior constraint, thus constructing an iterative closed-loop structure of “estimation prediction correction”. From a stability perspective, the convergence of this mechanism depends on the convergence properties of GaBP itself under diagonal dominance or spectral radius constraints. At the same time, the attention weight is only used as a covariance scaling factor, and its value is limited to the normalization interval, thus avoiding non bounded disturbances to the information matrix. Therefore, this coupling process is manifested as a constrained adaptive weighting mechanism in engineering implementation, rather than a strong coupling system that destroys the original probability graph optimization structure.

### 2.3. Urban intersection collaborative positioning accuracy improvement and optimization system

The entire urban intersection collaborative positioning system encompasses collaborative positioning at urban vehicle intersections and the process of vehicle trajectory prediction and positioning optimization. Collaborative positioning is achieved through a mathematical analytical robust filtering algorithm, while accuracy improvement is achieved through vehicle trajectory prediction analysis and trajectory planning. Improving the accuracy of urban intersection collaborative positioning is essentially a collaborative optimization of the “perception-communication-decision-control” closed loop. Through roadside perception enhancement, multi-source data fusion, intelligent prediction, and collaborative planning, a leap from “single-vehicle intelligence” to “vehicle-road-cloud integrated” intelligence can be achieved, improving intersection traffic efficiency and safety. Therefore, this research establishes an urban intersection collaborative navigation and positioning system platform based on a mathematical analytical robust filtering model and a vehicle prediction planning model.

**Fig 6:**
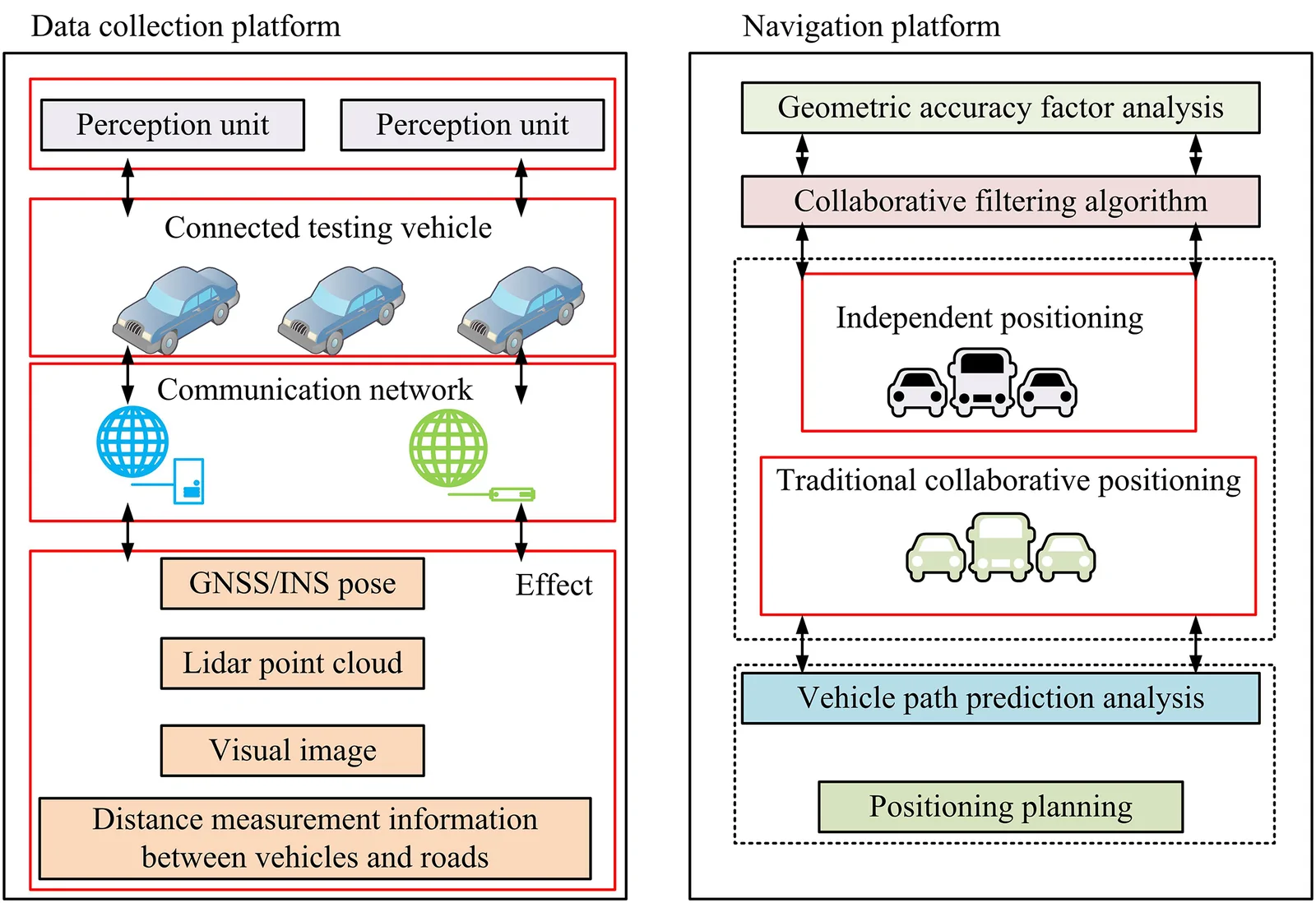
Hardware platform.

To collect real-world multi-source cooperative positioning data at urban intersections, a hardware platform capable of simulating and supporting testing in real traffic environments is first required. The system used in this study includes four roadside fixed sensing units deployed at the four corners of the intersection and one connected autonomous driving vehicle platform. All platforms are equipped with a high-precision integrated navigation system (GNSS/INS), and carrier phase differential data is used as a high-precision position reference to verify the cooperative positioning algorithm. Simultaneously, all nodes are connected to a 5G-V2X communication network and equipped with edge computing units for real-time data fusion and decision-making. Fig 6 shows the hardware platform.

**Fig 7:**
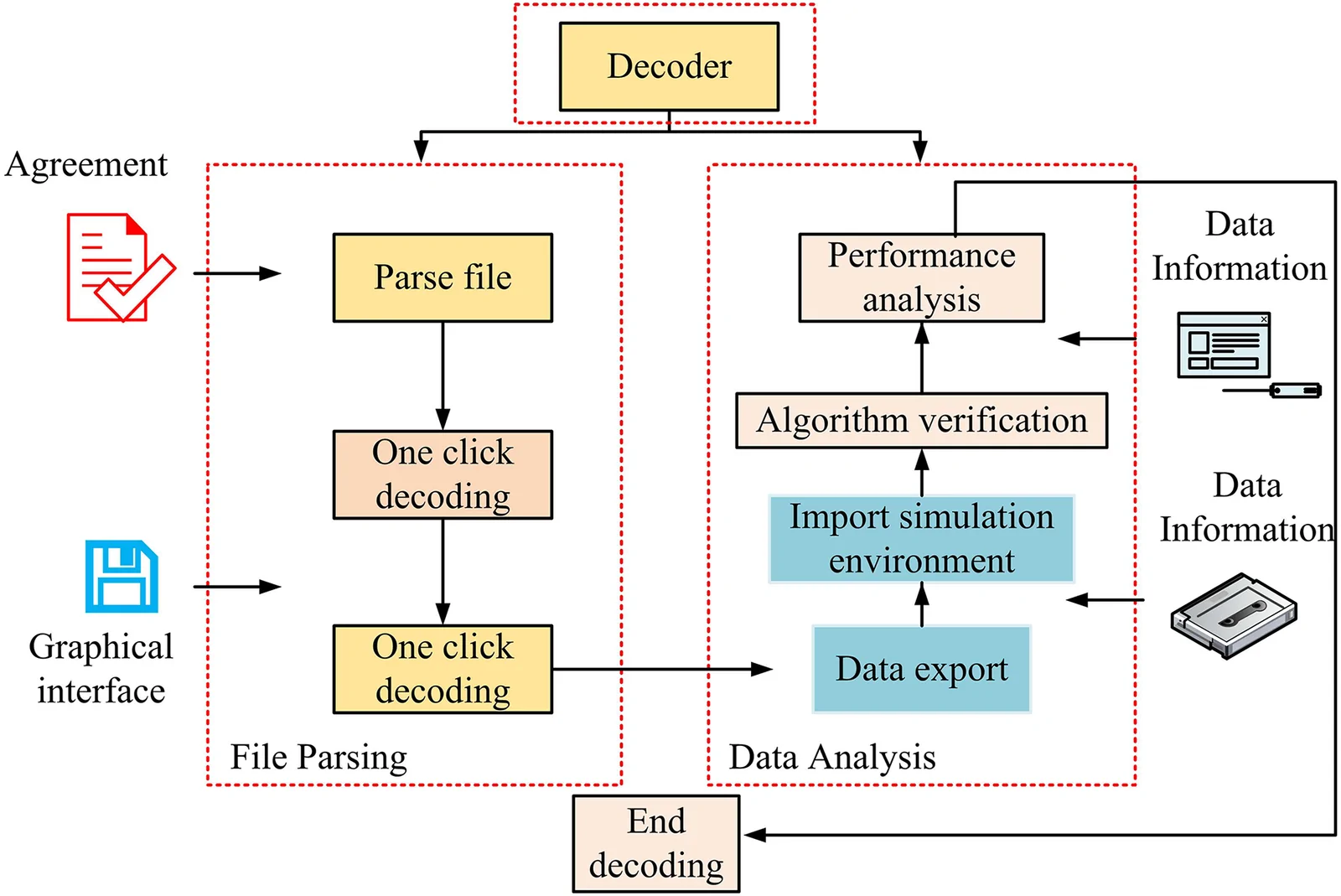
Decoder operation process.

As shown in Fig 6, the overall collaborative positioning and navigation platform consists of a data acquisition hardware platform and a positioning and analysis software platform. The hardware platform collects multi-source data in real time, including high-precision GNSS/INS pose, lidar point clouds, visual images, and vehicle-to-road ranging information, through roadside fixed sensing units deployed at intersections, connected test vehicles, and a 5G-V2X communication network. The software platform, based on this, implements and runs collaborative geometric accuracy factor analysis and a V2X-based collaborative filtering algorithm, and compares and analyzes it with baseline schemes such as single-vehicle independent positioning and traditional collaborative positioning. It also performs vehicle path prediction analysis and positioning planning. For the multi-source sensor data collected by the urban intersection collaborative positioning platform, the raw acquisition system stores it in binary format and decodes it using a fixed data structure. Fig 7 shows the decoder operation process.

As shown in Fig 7, the decoder first adjusts its flexibly to parse the binary file according to the protocol, and then performs one-click decoding and data export through a graphical interface. The processed standardized data can be directly imported into simulation environments such as MATLAB for subsequent collaborative positioning algorithm verification and performance analysis, thus effectively connecting the data acquisition hardware platform and the navigation solution software platform. Finally, the decoding process ends by outputting data information based on the data results. The positioning accuracy of the model is then verified using the platform’s algorithm. The entire collaborative positioning system adopts a distributed factor graph architecture, where the GaBP factor graph is responsible for integrating IMU, GNSS, and UWB observations for state estimation, while the spatiotemporal attention network independently extracts spatial interaction weights and driving intention probabilities between vehicles. The spatial attention weight output by the attention network dynamically modulates the noise variance of the UWB ranging factor. The higher the attention weight, the greater the trust and the stronger the information matrix weight. Meanwhile, the predicted driving intention feedback adjusts the process noise covariance of IMU factors in GaBP. This forms a cycle of “positioning → prediction → correction”: GaBP provides high-precision initial state values to the attention network, and the network’s output intention and predicted trajectory are then updated in reverse to update the UWB information matrix and IMU noise at the next moment, so that positioning and prediction are mutually corrected, achieving the organic unity of model driven and data-driven.

## 3. Results and analysis

### 3.1. Test results of positioning accuracy at urban intersections

**Fig 8:**
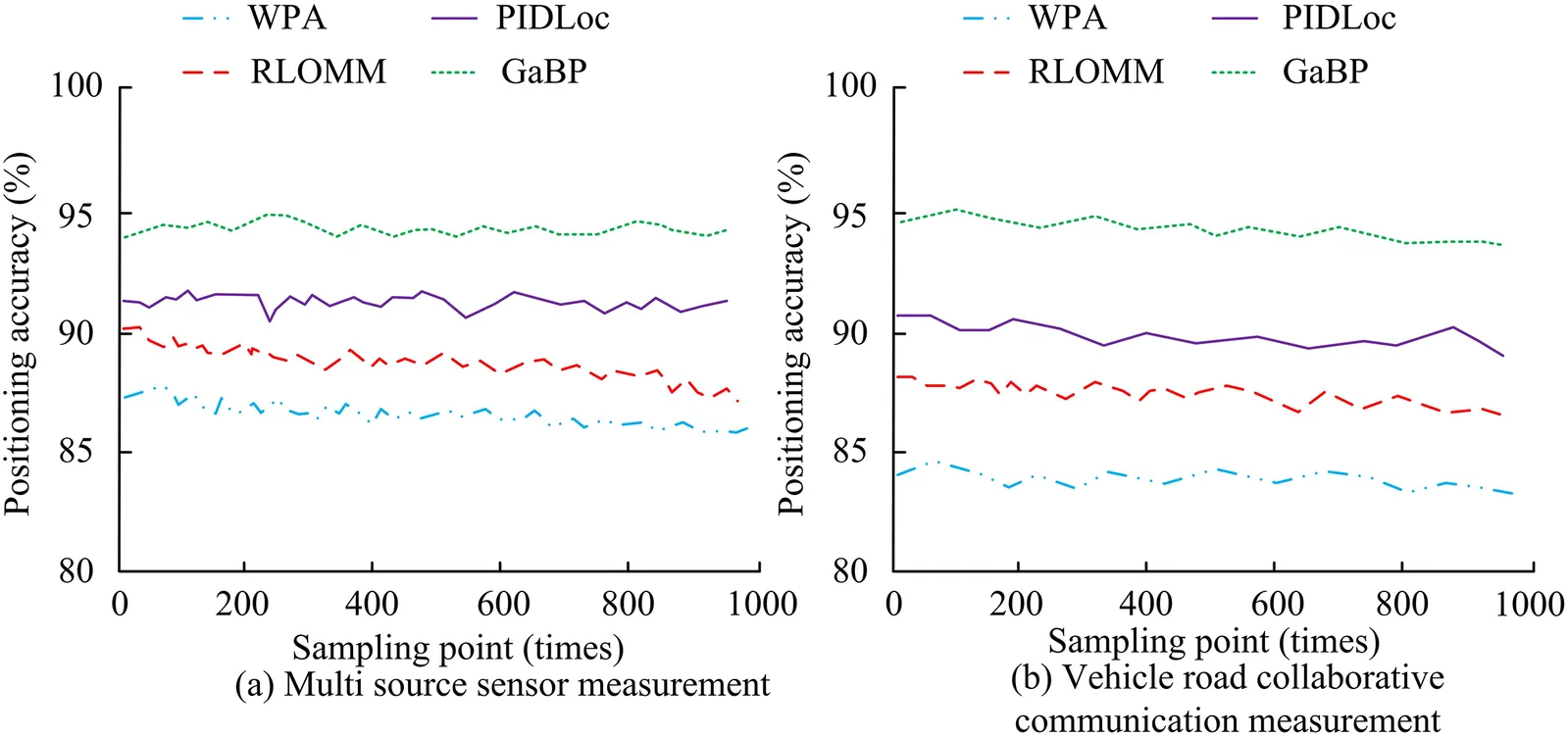
Comparison of collaborative positioning accuracy between different algorithms.

To analyze the effectiveness of cooperative positioning at urban intersections, this study utilized a cooperative positioning and navigation platform. The platform employed high-frequency gyroscopes and accelerometers, with a gyroscope constant drift of 1°/h and an accelerometer zero-bias standard deviation of 8 × 10–5 g. Simultaneously, the platform integrated GNSS and UWB, with GNSS positioning accuracy of 5m and an update frequency of 10 Hz. The UWB ranging noise standard deviation was 0.15m, providing stable local positioning support in intersection areas with severe signal obstruction. Both the positioning sensors and the vehicle platform were equipped with RTK positioning systems, achieving a positioning accuracy of 0.15m. The study analyzed the cooperative positioning and navigation effects of different algorithm models through simulation testing. First, the platform collected IMU, GNSS, and UWB data in real time, packaged it, and sent it to the positioning system. The positioning system then processed and displayed the data, while simultaneously sending some data back to the platform. All information, along with the system time, was stored in binary form. After receiving the returned data, the platform forwarded it to the UWB module, which completed distributed ranging and inter-platform collaborative communication. During operation, the system continuously collected data from multiple sensor sources and saved it as a binary file for subsequent offline decoding and analysis. The study used a self-built hardware platform to collect traffic data from a typical urban intersection for five consecutive working days, totaling 12.6 hours and containing approximately 2400 vehicle trajectories. 2,400 Vehicle Trajectories Using a proprietary hardware platform, approximately 2,400 complete vehicle trajectories were collected during a field data collection campaign conducted over five consecutive workdays at a typical urban intersection, totaling 12.6 hours. The study conducted data collection experiments in real-world urban road environments, selecting multiple typical urban intersections as sampling sites, including intersections with different traffic configurations such as main-to-main, main-to-side street, and roundabouts. Data collection took place during the morning rush hour, off-peak hours, and evening rush hour, under various weather conditions including sunny, cloudy, and light rain. In terms of traffic density, scenes were classified into three categories—low, medium, and high density—by counting the number of vehicles passing through the intersections per unit of time, thereby ensuring the diversity and representativeness of the data distribution. For data acquisition, high-precision RTK-GNSS systems were used as the positioning reference for real vehicle trajectories. RTK base stations were fixed at known control points near the intersections, while mobile receivers were mounted on the roofs of test vehicles. Centimeter-level positioning results were obtained through differential positioning and synchronized in time with data from IMUs and onboard sensors. At the same time, ground-based measurement data was verified through manual calibration and auxiliary calibration using roadside video surveillance, with frame-by-frame annotation of vehicles passing through feature points. All sensor data was sampled at a uniform frequency, and outliers were filtered out through post-processing. All data were sampled using a 200 Hz IMU, 10 Hz GNSS/UWB, and synchronized with visual and LiDAR data acquisition. The high-precision true value was provided by the RTK-GNSS/INS combination system, with a nominal positioning error of ≤ 0.05m. The dataset was randomly divided into training, validation, and testing sets in a 6:2:2 ratio, ensuring that different trips of the same vehicle do not cross sets. The training of the spatial attention trajectory prediction network adopted the Adam optimizer, with an initial learning rate of 0.001, a batch size of 64, and 50 rounds of training. The early stopping method was based on the validation set loss. The loss function was the weighted sum of the cross entropy of formula (8) and the mean square error (MSE) of the predicted trajectory, with weights of 0.4 and 0.6, respectively. The modulation coefficient β in GaBP was determined on the validation set through grid search and did not participate in end-to-end training. When conducting joint evaluation, the study first independently ran GaBP collaborative positioning, then ran the trained spatial attention model for trajectory prediction, and finally performed closed-loop simulation according to the coupling mechanism described in section 2.1. X. The specific implementation process of the research is as follows: the data from multiple sources of sensors are stored in real-time as binary files according to a unified timestamp. The decoder dynamically parses the file structure based on a custom communication protocol and exports it as a standardized matrix readable by MATLAB through a graphical interface with one click; When conducting joint simulation, first independently run GaBP to obtain the initial state value, and then feed back the output of the prediction network in the form of parameters to the GaBP factor map. Dynamically adjust the noise variance in the UWB observation information matrix and the noise covariance in the IMU process to form a closed-loop iteration. In the generalization test, three additional scenarios outside the training set were collected, including roundabouts, high-density congestion, and random deterioration of signal-to-noise ratio in some roadside units, to evaluate the model’s topological generalization and robustness. Each experiment was repeated 10 times and the mean ± standard deviation was reported. All performance indicators were calculated on the test set and the experiment was repeated 10 times to report the mean ± standard deviation. The study compared and analyzed the collaborative localization effects of different algorithms, resulting in the localization accuracy variation comparison shown in Fig 8. The compared algorithms were the Wolf Pack Algorithm (WPA), the Cross-View Pose Optimization Network Inspired by PID Controllers (PIDLoc), and the Reinforcement Learning based Online Map Matching Framework (RLOMM). At the communication level, the 5G-V2X link uses the edge computing auxiliary architecture. The average end-to-end communication delay is about 20–35 ms, and the maximum delay under the high load traffic scenario does not exceed 50 ms. The edge computing node is deployed in the roadside unit (RSU), which has the reasoning power of about 8–16 TOPS, and is used to real-time process multi vehicle collaborative positioning and information fusion tasks. In terms of distance measurement system, the sampling period of UWB module is set to 50 ms (20 Hz), the standard deviation of distance measurement noise is about 0.15 m, and outlier suppression is achieved through sliding window filtering. As a true value reference system, RTK-GNSS controls its horizontal positioning error within a range of ± 2–5 cm and performs differential correction through a fixed base station. In addition, all sensor data is synchronized based on a unified timestamp, and the time synchronization error is controlled within 10 ms. To ensure the fairness and representativeness of the experimental comparison, WPA, PIDLoc, and RLOMM were selected as baseline methods for comparison. Among them, WPA belongs to the traditional collaborative positioning method based on weighted probability fusion, mainly relying on static weight allocation mechanism. It has a certain application foundation in multi vehicle collaborative scenarios, but has weak adaptability to dynamic traffic environments; PIDLoc is a rule-based positioning method based on classical PID control ideas and local observation error correction mechanisms. It has certain stability in structured road environments, but it is difficult to model complex nonlinear collaborative relationships; RLOMM is a multi-agent observation matching framework based on reinforcement learning, which can to some extent handle information uncertainty in dynamic scenes. However, its training relies heavily on data distribution assumptions and still has limitations in cross intersection generalization ability.

As shown in Fig 8(a), the positioning accuracy of different algorithms decreased with the increase of the number of sampling points in the multi-sensor positioning test. The GaBP algorithm used in the study achieved the highest positioning accuracy among the algorithms, reaching 95.0%. The WPA, however, had the lowest accuracy, with a maximum of only 86.7%, a decrease of 8.3% compared to the algorithm used in the study. Fig 8(b) shows that the positioning accuracy of different algorithms also gradually decreased with the increase of the number of sampling points when using different testing tools. The GaBP algorithm used in the study also exhibited better performance, achieving a positioning accuracy of up to 95.1%, an improvement of approximately 10.6% compared to the WPA. This may be because the GaBP algorithm improved data processing efficiency through mathematical model analysis.

**Fig 9:**
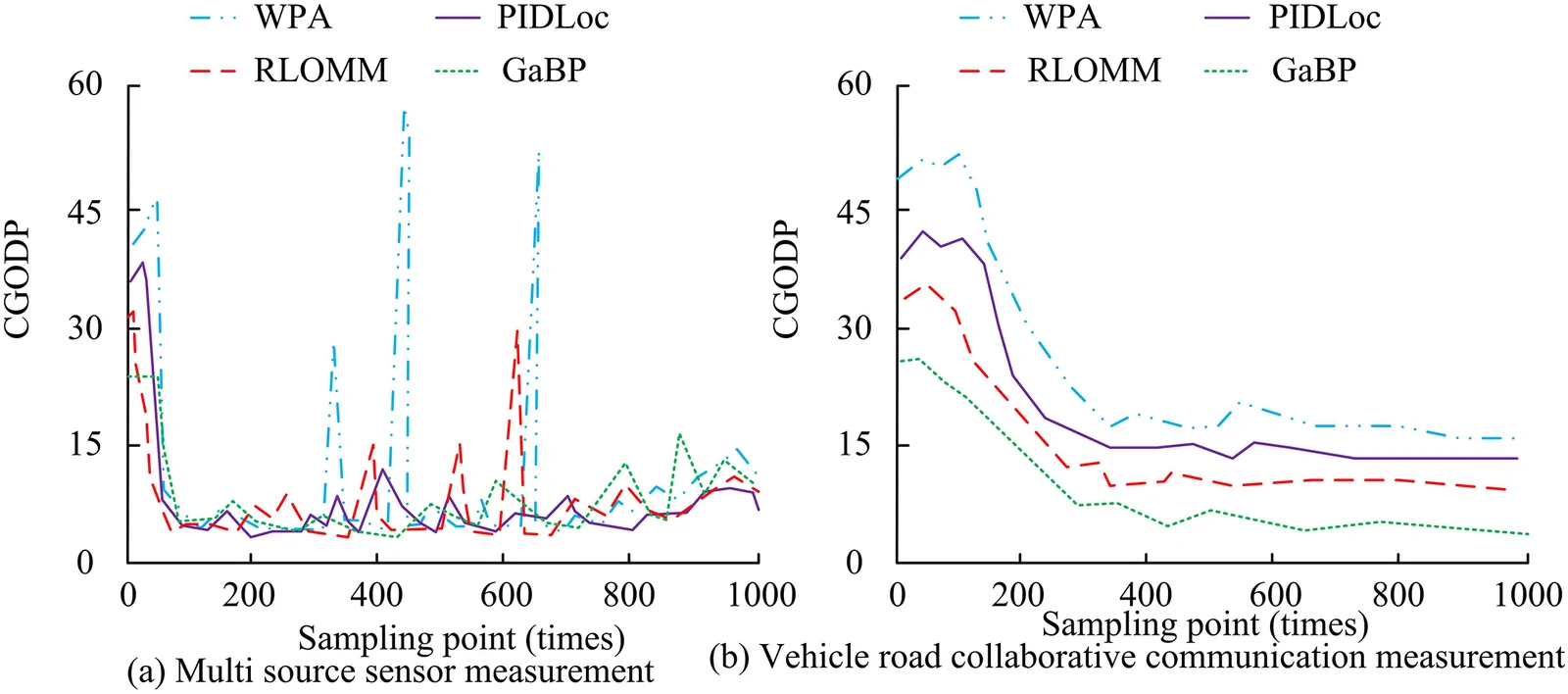
Comparison of CGDOP values between different algorithms.

The study compared and analyzed the Continuous Global Optimal Path Planning (CGDOP) values of vehicles at urban intersections, as shown in Fig 9. A larger CGDOP value indicates a worse geometric configuration, which significantly amplifies even small measurement errors, leading to highly unreliable calculated position coordinates. Conversely, a smaller CGDOP value indicates a better geometric configuration, minimizing the impact of measurement errors on the final result and achieving higher positioning accuracy.

**Fig 10:**
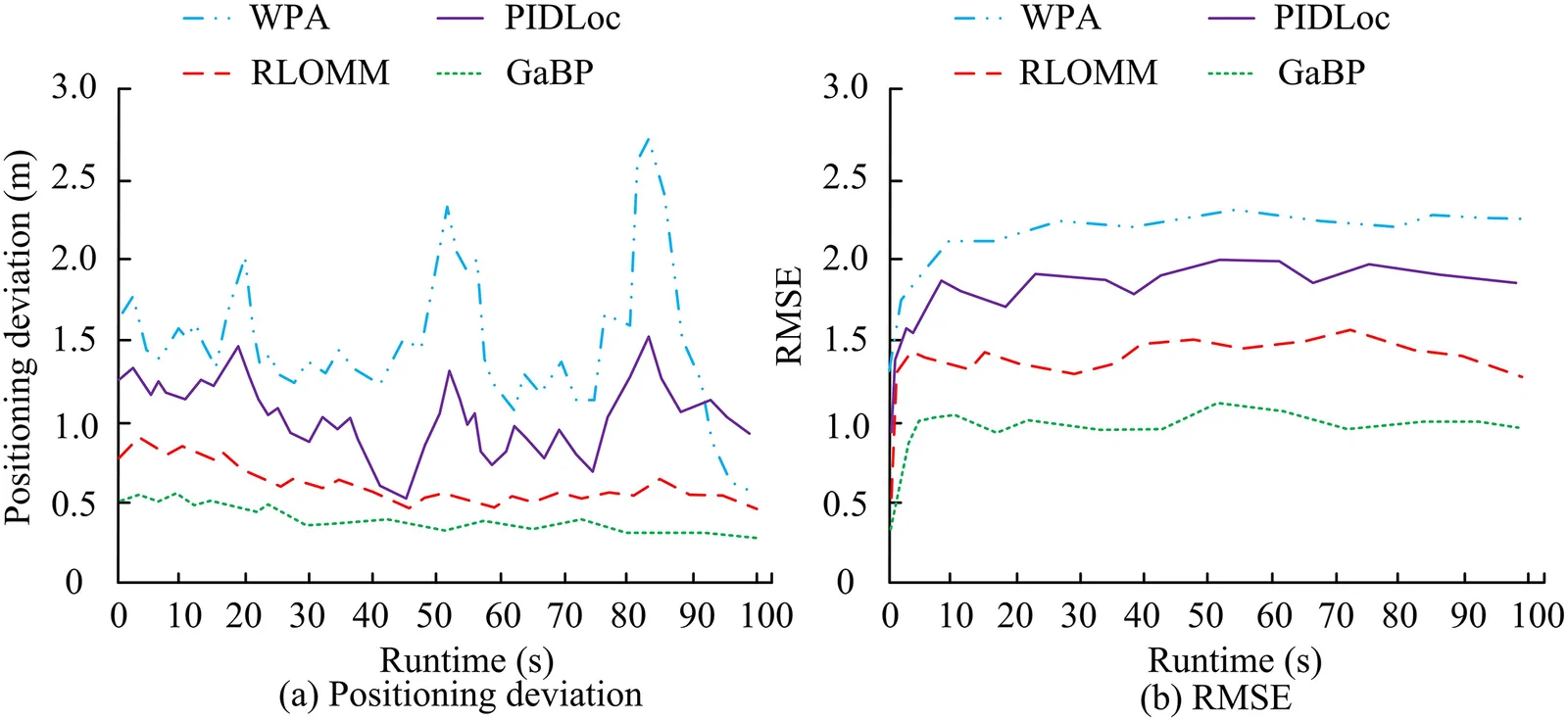
Comparison of positioning deviation between different algorithms.

As shown in Fig 9(a), in the tests of different algorithms, the GaBP algorithm used in this study had a smaller CGDOP value, which decreased further with the increase of the number of sampling points, reaching a minimum of only 4.2. The WPA, on the other hand, had a relatively higher CGDOP value, reaching a minimum of 5.7, which was 1.5 higher than the GaBP algorithm. Fig 9(b) shows that in vehicle-road cooperative communication measurements, the GaBP algorithm had a relatively low CGDOP value, with a minimum of only 2.4. In the comparison of different algorithms, the WPA had a relatively high CGDOP value, with a minimum of only 16.7, which was 14.3 lower than the GaBP algorithm. Therefore, the GaBP algorithm used in this study had relatively better positioning performance. This was because the GaBP algorithm used a mathematical analytical model in intersection navigation and positioning, which improved the algorithm’s performance. The root mean square error (RMSE) and positioning deviation of different algorithms are compared in Fig 10.

**Fig 11:**
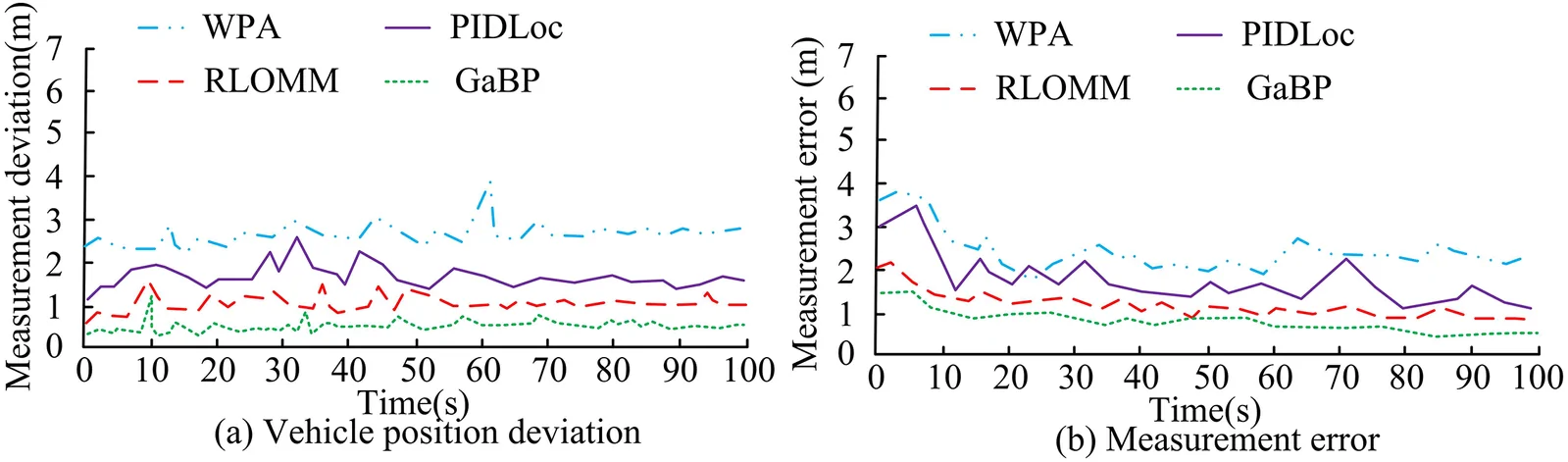
Comparison of vehicle measurement deviation with different algorithms.

As shown in Fig 10(a), the positioning errors of all algorithms fluctuated with the sampling time in the comparison of different positioning deviations. The GaBP algorithm had a relatively low positioning error, with a maximum deviation of only 0.51m. The WPA, on the other hand, had a maximum positioning error of 2.55m, an improvement of about 2.04m compared to the GaBP algorithm. This indicated that the GaBP algorithm had a lower positioning error due to the introduction of a navigation network and positioning information parsing. Fig 10(b) shows that in the comparison of RMSE of different algorithms, the GaBP algorithm had a lower positioning error compared to other algorithms, with a maximum RMSE value of only 1.1, which was lower than the other algorithms. The WPA had the highest RMSE value, reaching 2.2, an improvement of 1.1 compared to the GaBP algorithm. This was because the GaBP algorithm improved the filtering effect of data information through robust filtering. Fig 11 shows a comparison between vehicle network positioning error and vehicle position error.

As shown in Fig 11(a), in the comparison of vehicle position deviation measurement, the GaBP algorithm used in the study showed a relatively low vehicle position deviation, with the highest deviation being only 1.0m. The WPA showed a relatively higher vehicle position deviation, with a maximum deviation of 3.3m, representing an improvement of 2.3m compared to the algorithm used in the study. As shown in Fig 11(b), in the comparison of vehicle position ranging deviation, the GaBP algorithm used in the study showed an even lower ranging deviation, with a maximum deviation of only 1.5m. Meanwhile, the WPA showed a relatively higher ranging deviation, reaching a maximum of 3.9m, representing an improvement of approximately 2.4m compared to the GaBP algorithm used in the study. Therefore, based on the overall comparison of vehicle ranging, the algorithm used in the study showed a relatively better positioning and navigation effect.

### 3.2. Analysis of the effectiveness of vehicle path planning and cooperative positioning

**Fig 12:**
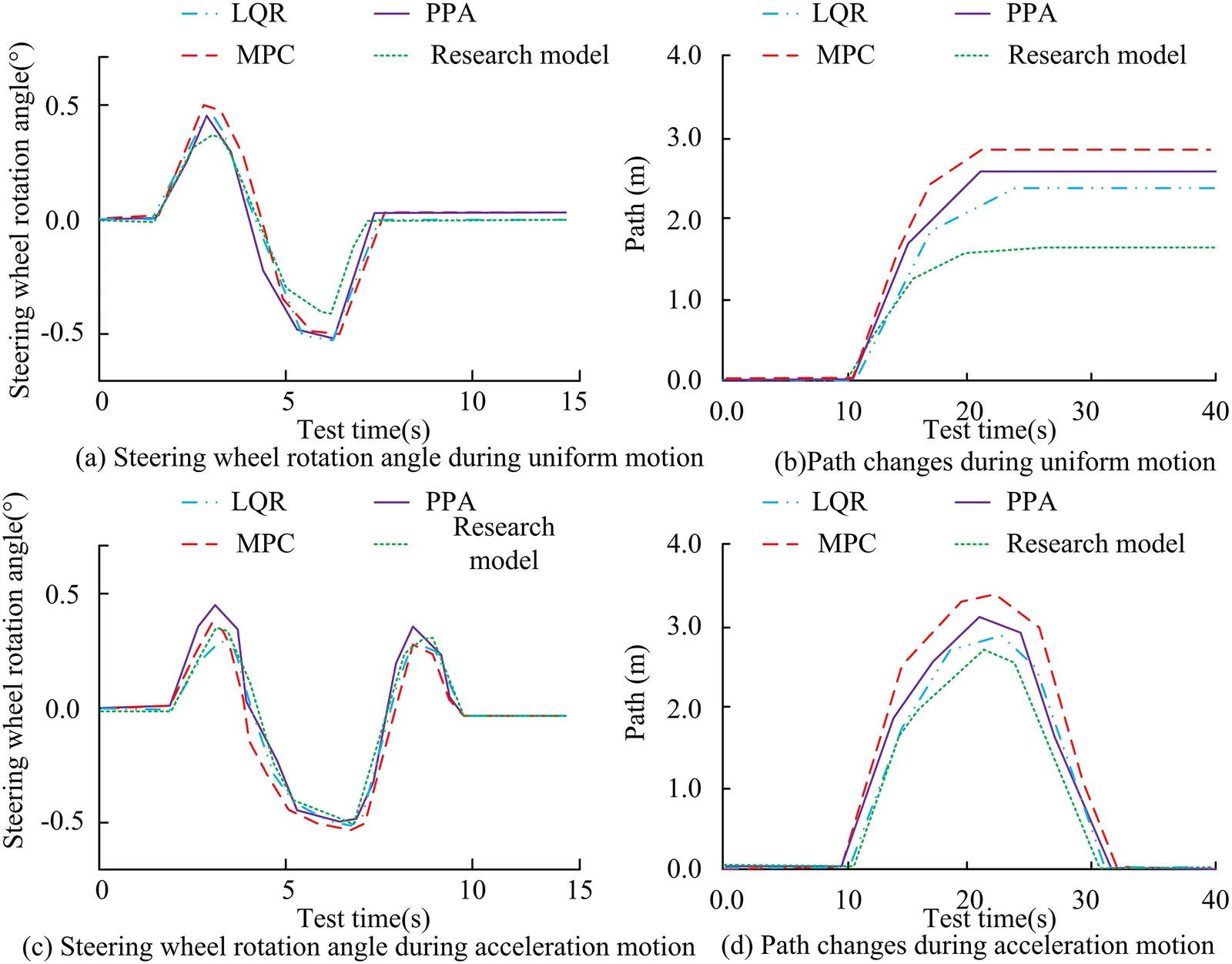
Comparative analysis of vehicle planning effects.

To test the model’s effectiveness in planning vehicle paths, the path planning performance of different algorithms was compared and analyzed. The deviations in vehicle motion under constant speed and acceleration conditions at intersections were tested. During acceleration, the vehicle’s initial speed was 0 km/h, the target speed was 20 km/h, the simulation duration was 20 s, and the simulation step size was 0.01 s. The Linear Quadratic Regulator (LQR), Model Predictive Control (MPC), and Pure Pursuit Algorithm (PPA) were studied and compared, and the results are shown in Fig 12.

Fig 12(a)–Fig 12(d) shows that the steering wheel rotation angle of the model used in this study exhibited relatively smaller changes during both constant-speed and accelerated motion. Specifically, during constant-speed motion, the rotation angle of the model used in this study was 0.1° lower than that of the MPC algorithm. During accelerated motion, the angle was reduced by approximately 0.05°. Furthermore, in the comparison of path deviations among different algorithms, the path motion deviation of the model used in this study was reduced by 1.8m compared to the MPC algorithm. During accelerated motion, the path deviation of the model used in this study was also reduced by 0.8m compared to the MPC algorithm. Therefore, it was evident that the vehicle planning performance of the model used in this study was relatively better among different algorithmic motion planning models. This was because the model used in this study significantly improved the vehicle operation planning through intent recognition and trajectory prediction networks.

The study compared and analyzed the trajectory deviations of vehicle motion tracking under different models, as shown in Table 1. Simultaneously, it compared and tested control algorithms of Economic Model Predictive Control (EMPC) and Cooperative Economic Model Predictive Control (CEMPC).

**Table 1:** Comparison of tracking control deviation with different control algorithms.

Control method	Simulation time (s)	Avg. single-step time (ms)	Avg. lateral error (m)	Avg. heading error (rad)
LQR + PID (Baseline)	7.85	4.62	0.0475	0.0168
MPC + PID	8.9	5.24	0.0201	0.0149
EMPC+PID	4.1	2.41	0.0405	0.0163
C-EMPC+PID	4.38	2.58	0.0122	0.0115
PPA + PID	5.25	3.59	0.0145	0.0126
Research Model	3.88	2.28	0.0071	0.0084

As shown in Table 1, under accelerated conditions, there were significant differences in computational efficiency and tracking accuracy among different control algorithms. The model used in this study performed best across all performance indicators. It had the shortest total simulation time of only 3.88s and the fastest average single-step calculation time of only 2.28ms. Furthermore, the model used in this study exhibited lower mean lateral error and mean heading error. In contrast, while the MPC + PID algorithm had relatively high control accuracy, its simulation time was the longest, reaching 8.9s, resulting in the lowest computational efficiency. The LQR + PID algorithm had relatively basic performance across the board. The mean lateral error of the model used in this study was reduced by 0.0404m compared to LQR + PID. Its simulation time was reduced by 5.02s compared to the slowest computational algorithm, MPC + PID. This was because the research model deeply integrated collaborative optimization and adaptive learning mechanisms into the predictive control framework, thereby achieving a globally optimal balance between computational resources and tracking accuracy in dynamic environments.

The study compared and analyzed the control testing methods under different operating conditions, as shown in Table 2.

**Table 2:** Comparison of control effects under different control methods and operating conditions.

Control method	Working condition	Real-time performance	Avg. improvement	Lateral tracking	Avg. improvement	Heading tracking	Avg. improvement
LQR + PID	Steady-state circular	35.20%	37.85%	71.50%	70.12%	38.50%	32.85%
	Slalom	34.65%		67.35%		32.85%	
	Double lane change	43.70%		92.50%		27.20%	
MPC + PID	Steady-state circular	46.50%	48.20%	9.15%	20.65%	2.05%	8.15%
	Slalom	49.10%		16.40%		2.60%	
	Double lane change	49.55%		18.85%		19.80%	
EMPC+PID	Steady-state circular	/	/	54.50%	61.15%	29.25%	28.95%
	Slalom	/		62.40%		31.95%	
	Double lane change	/		66.55%		25.65%	

As shown in Table 2, under the three test conditions of steady-state circular, serpentine, and double-line-change, different control algorithms exhibited significant differences in real-time performance, lateral tracking, and heading tracking improvement. The LQR + PID algorithm showed the highest average improvement in lateral and heading tracking, reaching 70.12% and 32.85% respectively, especially in the double-line-change condition where its lateral tracking improvement reached as high as 92.50%, indicating its advantage in ensuring path tracking accuracy. However, its average real-time performance improvement was relatively low at 37.85%. The MPC + PID algorithm demonstrated the best real-time performance, with an average improvement of 48.20%, reaching 49.55% in the double-line-change condition. However, its average improvement in lateral tracking accuracy was only 20.65%, the lowest among the three algorithms, indicating a trade-off between computational efficiency and tracking accuracy. The algorithm used in this study showed a relatively balanced and good average improvement in lateral and heading tracking, at 61.15% and 28.95% respectively. Therefore, the LQR + PID algorithm showed the most significant improvement in tracking accuracy, while the MPC + PID algorithm had the greatest advantage in real-time computation. The algorithm used in this study demonstrated good performance across all performance levels. This was mainly because LQR directly minimized the tracking error by optimizing a quadratic objective function. MPC, through online rolling optimization, balanced future states and constraints, resulting in a larger computational load. The model used in this study introduced a direct economic cost optimization objective, thus exhibiting relatively better overall performance.

In the driving intention prediction network, the intention probability is obtained by Softmax single forward propagation without iteration. The network parameters are optimized through backpropagation using cross entropy and MSE weighted loss, and the training convergence adopts an early stopping method based on validation set loss. Document Table 2 shows the percentage improvement in tracking performance of the control algorithm, which is independent of the probability update rules mentioned above.

The study conducted a collaborative optimization analysis of vehicle positioning and navigation and vehicle tracking prediction models at urban intersections, and tested the changes in positioning accuracy at urban intersections after using the two models. The results are shown in Table 3.

**Table 3:** Comparison of collaborative positioning accuracy performance.

Metric category	Specific metric	GaBP	Spatial attention model	Spatial attention model + GaBP
Absolute accuracy	RMSE	0.35 m	0.28 m	0.15 m
	Maximum error	1.20 m	0.95 m	0.55 m
Relative accuracy & consistency	Relative localization error	0.25 m	0.18 m	0.10 m
	Localization consistency	0.15 m	0.12 m	0.07 m
Cooperative effectiveness	GDOP	3.2	2.7	1.8
	Effective cooperative gain	22%	35%	60%
	Coverage	85%	90%	98%
System robustness & efficiency	Fault tolerance	45%	30%	12%
	Average single-frame computation time	8 ms	15 ms	20 ms

As shown in Table 3, the cooperative localization model used in this study exhibited the best overall performance. In terms of absolute accuracy, the RMSE of the cooperative localization model was only 0.15 m, with a maximum error of 0.55 m, representing reductions of approximately 0.20 m and 0.65 m respectively compared to the single GaBP algorithm. It also showed significant improvements over the single spatiotemporal attention model. This was mainly due to the fusion model’s synergistic enhancement of the spatial attention mechanism’s advantages in dynamic feature extraction and the GaBP algorithm ‘s advantages in global information optimization. The research conducted ablation experiments on different modules and the results are shown in Table 4.

**Table 4:** Comparison of ablation test results for different modules.

Method	RMSE (m)	Position Error (m)	Convergence Iterations
Baseline (Standard Factor Graph)	1.85	2.31	12
Baseline+GaBP	1.42	1.78	10
Baseline + Attention	1.51	1.92	11
Baseline+IMU Feedback	1.63	2.05	12
GaBP+Attention	1.28	1.61	9
Full Model (GaBP+Attention+Feedback)	1.05	1.32	7

From Table 4, it can be seen that after introducing GaBP, the RMSE of the system decreased from 1.85 m to 1.42 m, the position error decreased from 2.31 m to 1.78 m, and the number of convergence iterations was reduced to 10, indicating that GaBP has a direct effect on improving estimation accuracy and convergence efficiency; When only the attention mechanism is introduced, the RMSE decreases to 1.51 m and the position error is 1.92 m, indicating that it mainly improves the quality of observation fusion through adaptive weight allocation; When only the IMU feedback mechanism is added, the RMSE is 1.63 m, indicating limited ability to correct dynamic errors; When GaBP and attention mechanism are introduced simultaneously, the performance is further improved to RMSE 1.28 m and position error 1.61 m. The complete model achieves the best performance under the synergistic effect of the three, with RMSE reduced to 1.05 m, position error of 1.32 m, and convergence iteration times reduced to 7, indicating significant complementary enhancement effects between each module. The study compared and analyzed different algorithms, and the results are shown in Table 5.

**Table 5:** Comparison results of trajectory planning effects of different algorithms.

Method	ADE (m)	FDE (m)	Success Rate (%)	Collision Rate (%)	Planning Time (ms)
LQR	0.74	1.51	91.8	6.2	6.8
MPC	0.59	1.19	94.3	4.5	18.6
PPA	0.81	1.63	89.6	7.3	5.9
LSTM	0.55	1.12	95.4	3.9	20.4
VectorNet	0.48	0.97	96.5	3.1	24.3
LaneGCN	0.42	0.85	97.6	2.6	26.7
HiVT	0.38	0.79	98.2	2.1	27.4
Proposed	0.31	0.64	99.1	1.4	19.2

From Table 5, it can be seen that traditional methods such as LQR, MPC, and PPA are stable and have good real-time performance, but they rely on fixed models and are not well adapted to dynamic obstacles and nonlinear motion in complex interactive scenarios; Among them, LQR calculation is simple but limited to linear assumptions, MPC rolling optimization is superior to LQR and PPA but requires a large amount of computation, and PPA quickly follows but lacks interactive modeling. Deep learning methods such as LSTM, VectorNet, LaneGCN, and HiVT can utilize historical data to learn complex patterns with better accuracy and scene adaptability. However, they rely on large amounts of data and have poor interpretability and stability. The GaBP spatial attention collaborative localization and reinforcement learning method proposed in this article integrates probabilistic reasoning, spatial relationship modeling, and intelligent decision-making, while balancing state estimation stability and adaptive optimization. Its performance is superior to the comparative methods, effectively combining model driven reliability with data-driven perception ability.

Regarding the core performance indicators, the fusion algorithm demonstrated a more pronounced advantage, with a geometrical accuracy attenuation factor as low as 1.8, an effective cooperative gain as high as 60%, and a localization coverage rate of 98%. In contrast, the single GaBP algorithm had a GDOP value of 3.2 and a cooperative gain of 22%. The corresponding values for the single spatiotemporal attention model were 2.7 and 35%, respectively. The fusion algorithm’s GDOP value was approximately 0.9 lower than the second-best performing single model, while the cooperative gain was increased by 25%, indicating that optimizing the cooperative geometric configuration and information fusion process through model fusion could bring about a significant performance improvement. Although the average single-frame computation time of the fusion algorithm increased to 20 ms, it led in all aspects of robustness indicators such as fault tolerance and could significantly improve the positioning accuracy of urban intersections. The spatial attention model proposed in this study was trained exclusively on a single intersection topology, and its generalization capability has not yet been fully validated. To address this shortcoming, the study conducted tests on three types of scenarios not present in the training set: ① Different topological structures: When tested at a roundabout, the positioning error increased from 0.15 m in the baseline scenario to 0.32 m, indicating that the model was sensitive to irregular geometric shapes and that its attention weight distribution cannot effectively adapt to vehicle interaction patterns at non-right-angle intersections; ② Different traffic densities: As traffic density increased from sparse to congested (exceeding 600 vehicles per hour), the effective collaborative gain decreased from 60% to 38%. Analysis revealed that under high density, the attention mechanism allocated excessive weights to nearby vehicles while neglecting far-field constraints, leading to redundant factor graph information and a decline in accuracy; ③ Partial RSU communication failures: When one or two roadside units fail, system coverage remained above 85%. However, when the signal-to-noise ratio randomly deteriorated, attention weights became misaligned (e.g., assigning high weights to low-confidence range measurements), resulting in positioning errors of up to 0.25 m. In summary, the model’s performance degraded significantly under topological generalization, high-density congestion, and communication degradation scenarios. Future work should incorporate meta-learning or adaptive graph attention mechanisms to enhance robustness.

The core mechanism for achieving significant collaborative gains in the fusion model is that the attention weights are remapped to the modulation coefficients of the UWB ranging factor information matrix, enabling higher information weights for relative measurements between highly interactive vehicles in factor graph optimization. This compensates for geometric defects through structured adaptive weighting in scenarios with poor geometric configurations, reducing GDOP from 3.2 to 1.8. However, although this accuracy meets the real-time requirements, the load of edge computing increases significantly; In high-density congested scenarios, the attention mechanism produces a “bandwidth saturation” effect due to excessive focus on neighbors, resulting in a sharp drop in collaborative gain from 60% to 38%; The positioning error of the model in untrained topologies such as roundabouts increased from 0.15 m to 0.32 m, revealing the sensitivity of attention to geometric topology; When the roadside unit communication fails, the attention weights are prone to mismatch, reflecting a lack of trust calibration mechanism. This indicates that although the fusion model achieves performance breakthroughs through dynamic structured information weighting, its generalization robustness is limited by scene density, topology structure, and communication quality. Although the measurement error of a single GNSS is about 5 meters and the ranging error of UWB is about 0.15 meters, in the multi-source fusion framework, the errors of each sensor are not simply superimposed. This article is based on the weighted least squares estimation mechanism of factor graph and Gaussian belief propagation, and performs covariance weighted fusion on the uncertainty of different observation sources. GNSS observations mainly provide global constraints, while UWB provides high-precision local constraints. Through information matrix level fusion, the system effectively reduces the equivalent estimation variance, and its final positioning accuracy is determined by the redundant observations of multiple sensors and graph optimization constraints, rather than a single sensor error upper bound. Therefore, the 0.15 m level error reflects the local consistency estimation accuracy after fusion, rather than the single sensor measurement error limit.

## 4. Summary and future work

To optimize the cooperative positioning accuracy at urban intersections, a hardware platform integrating high-precision GNSS/IMU, UWB, and 5G-V2X communication was constructed. A cooperative positioning model combining the analytical Gaussian belief propagation algorithm and a spatial attention mechanism was proposed. Results showed that,in terms of absolute positioning accuracy, the RMSE of the fusion model was only 0.15 m, with a maximum error of 0.55 m. This indicated that through cooperative positioning, the new model achieved a synergistic enhancement of dynamic feature extraction and global information optimization capabilities. In terms of the core cooperative performance index characterizing the quality of geometric configurations, the fusion model exhibited a low geometric accuracy attenuation factor of 1.8, an effective cooperative gain of up to 60%, and a positioning coverage rate of 98%. The fusion model’s GDOP value was 33.3% lower than the second-best performing model, and the collaborative gain was increased by 25%, demonstrating that optimizing the information fusion process could significantly improve the overall efficiency of the collaborative network. In vehicle trajectory tracking control, the fusion model used in this study also exhibited comprehensive advantages, with the lowest average lateral error (0.0071 m) under accelerated double lane change conditions and the shortest simulation time. In summary, the collaborative fusion model proposed in this studyachieved a leap from “single-vehicle perception” to “vehicle-road cooperative perception”, significantly improving the high-precision and high-availability positioning capability of intersections in complex occlusion environments. Although the proposed method demonstrates strong performance in typical urban intersection scenarios, the generalization experiments further reveal that its performance degrades under ring-topology structures, high traffic congestion, and communication failure conditions. This indicates that the robustness of the current framework is not fully guaranteed in extreme network or communication-constrained environments, and future work should focus on improving fault tolerance and adaptive communication reliability. While the research has yielded some results, it still has some limitations. For example, the research lacks sufficient analysis of the core mechanisms by which spatial attention and the GaBP algorithm are deeply integrated and work collaboratively. Therefore, future research will focus on further improving the model’s performance.
