# Supplementary material for: Improving the accuracy of distributed cooperative positioning at urban intersections based on mathematical analysis and robust filtering
Source: PLoS One. 2026 Sep 24;21(9):e0358244. doi: 10.1371/journal.pone.0358244 (PMC13606937; doi:10.1371/journal.pone.0358244)
Supplement: Supplementary file 1 [file pone.0358244.s001.doc]

Reviewer #1: The paper presents a study on improving the accuracy of distributed cooperative positioning at urban intersections based on mathematical analysis and robust filtering. Based on the critical points below, I recommend major revision before the manuscript can be considered for publication.

1. The claimed theoretical innovation of bidirectionally coupling Gaussian belief propagation with spatial attention weights is not rigorously derived. No mathematical proof or convergence analysis is provided to demonstrate that the closed-loop feedback of driving intent into IMU noise covariance is stable or optimal.

Reply: Thank you for your review comments. The mathematical proof and the convergence analysis of the proposed method have been provided, as shown below:

**The mathematical proof:**

GaBP is essentially equivalent to iteratively solving the following quadratic objective function under a Gaussian diagram model.

$\min_{x} \frac{1}{2}x^{T}Jx-h^{T}x$ (12)

Where $J$ is the information matrix. In this method, spatial attention weights are introduced to modulate the observed covariance.

$R^{;}=\alpha R$ (13)

$\alpha$ represents spatial attention weight. The information matrix modulated with attention is denoted as.

$J^{,}=J_{GaBP}+\triangle J(\alpha)$ (14)

Where $\triangle J(\alpha)$ is a bounded perturbation term. The overall iterative update can be regarded as a bounded perturbation system based on the contraction mapping.

$x^{t+1}=F(x^{t})+\vartheta(\alpha)$ (15)

When $F(x^{t})$satisfies the contraction condition and the perturbation term $\vartheta(\alpha)$ is bounded, according to Banach fixed point theory, the system iteration still converges to a stable solution in the neighborhood without divergence. When the information matrix satisfies the strict diagonal dominance condition, the GaBP algorithm can ensure convergence. Establish an overall information matrix

$\triangle=D+R$ (16)

$D$ represents the diagonal matrix, and $R$ represents the diagonal correlation matrix. The matrix satisfies the Walk Summability condition. Further define the GaBP iteration matrix

$T=-D^{-1}R$ (17)

The convergence condition is:

$\rho(T)<1$ (18)

$\rho(T)$ represents the spectral radius of the matrix. After introducing spatial attention modulation.

$\beta<\frac{1}{\left\|A\right\|}$ (19)

According to Banach fixed point theorem, the iterative process.

$J^{(k+1)}=F(J^{(k)})$ (20)

There exists a unique fixed point.

$J^{(*)}=F(J^{(*)})$ (21)

**The convergence analysis:**

The GaBP model with added spatial attention still maintains convergence stability and will not cause information propagation divergence due to dynamic weight adjustment. Previous studies have shown that GaBP can achieve stable estimation performance when satisfying diagonal dominance and spectral radius constraints. For example, Murai et al. used Gaussian belief propagation to achieve distributed synchronous positioning, which maintains stable convergence performance even in the presence of communication noise[34].

**The comparision of the theoretical boundaries of the pure GaBP and the pure spatial attention model:**

The bidirectional coupling framework of Gaussian belief propagation (GaBP) and spatial attention mechanism proposed in the study is essentially an adaptive information adjustment mechanism based on a structured probability graph model. This method is not strictly a globally optimal analytical solution, but rather achieves dynamic recalibration of information weights in the factor graph by introducing attention weights into the observation covariance adjustment term. At the same time, the driving intention output by the trajectory prediction module is fed back to the IMU process noise update term as a prior constraint, thus constructing an iterative closed-loop structure of "estimation prediction correction". From a stability perspective, the convergence of this mechanism depends on the convergence properties of GaBP itself under diagonal dominance or spectral radius constraints. At the same time, the attention weight is only used as a covariance scaling factor, and its value is limited to the normalization interval, thus avoiding non bounded disturbances to the information matrix. Therefore, this coupling process is manifested as a constrained adaptive weighting mechanism in engineering implementation, rather than a strong coupling system that destroys the original probability graph optimization structure.

2. The technical novelty is overstated. Integrating existing GaBP with a standard spatiotemporal attention network does not constitute a fundamentally new method, and similar factor graph-based collaborative positioning frameworks have been reported in prior literature.

Reply: Thank you for your review comments. The innovations of our work have been revised, as shown below:

(1) Theoretical Innovation: The innovation of this study lies primarily in the collaborative modeling of traditional Gaussian belief propagation and spatio-temporal attention mechanisms within a unified factor graph framework, and in achieving bidirectional adaptive updating between observed and prior information by introducing an uncertainty-based weight modulation strategy. This method differs from existing collaborative localization methods based on fixed weights or unidirectional fusion, enhancing the flexibility and robustness of information fusion in complex dynamic environments.

(2) Technical Innovation: A distributed collaborative computing framework with low communication overhead is constructed, integrating the GaBP robust filtering algorithm with a spatiotemporal attention-based trajectory prediction network within a single factor graph optimization framework. A hardware verification platform is designed that integrates roadside sensing units, connected test vehicles, and 5G-V2X communication. A complete software workflow is developed, ranging from binary data decoding to standardized output, enabling real-time acquisition, collaborative processing, and closed-loop verification of multi-source heterogeneous sensors .

(3) Application Innovation: Addressing the practical challenges of severe GNSS signal obstruction and complex, dynamic traffic flows at urban intersections, this work provides a distributed collaborative positioning solution characterized by high accuracy, high robustness, and high coverage. Field test results demonstrated that the proposed fusion model achieved a positioning RMSE of only 0.15 m, an effective collaborative gain of up to 60%, and a positioning coverage rate of 98%, with a geometric accuracy degradation factor as low as 1.8. This successfully bridges the gap from single-vehicle intelligent perception to vehicle-road collaborative swarm intelligence, providing critical technical support for reliable perception at smart intersections and connected autonomous driving.

3. The experimental validation lacks a proper ablation study. It is impossible to determine whether the performance gains come from the GaBP algorithm, the attention mechanism, or their coupling because no separate evaluation of each component under identical conditions is presented..

Reply: Thank you for your review comments. The ablation experiments have been provided, as shown below:

The research conducted ablation experiments on different modules and the results are shown in Table 4.

Table 4 Comparison of ablation test results for different modules

| Method | RMSE (m) | Position Error (m) | Convergence Iterations |
| --- | --- | --- | --- |
| Baseline (Standard Factor Graph) | 1.85 | 2.31 | 12 |
| Baseline+GaBP | 1.42 | 1.78 | 10 |
| Baseline + Attention | 1.51 | 1.92 | 11 |
| Baseline+IMU Feedback | 1.63 | 2.05 | 12 |
| GaBP+Attention | 1.28 | 1.61 | 9 |
| Full Model (GaBP+Attention+Feedback) | 1.05 | 1.32 | 7 |

From Table 4, it can be seen that after introducing GaBP, the RMSE of the system decreased from 1.85 m to 1.42 m, the position error decreased from 2.31 m to 1.78 m, and the number of convergence iterations was reduced to 10, indicating that GaBP has a direct effect on improving estimation accuracy and convergence efficiency; When only the attention mechanism is introduced, the RMSE decreases to 1.51 m and the position error is 1.92 m, indicating that it mainly improves the quality of observation fusion through adaptive weight allocation; When only the IMU feedback mechanism is added, the RMSE is 1.63 m, indicating limited ability to correct dynamic errors; When GaBP and attention mechanism are introduced simultaneously, the performance is further improved to RMSE 1.28 m and position error 1.61 m. The complete model achieves the best performance under the synergistic effect of the three, with RMSE reduced to 1.05 m, position error of 1.32 m, and convergence iteration times reduced to 7, indicating significant complementary enhancement effects between each module.

4. The latest advances on vehicle positioning and V2X should be reviewed for completeness. For instance, A framework for real-time vehicle tracking in large-scale roadside sensor networks; Optical communication based V2V for vehicle platooning.

Reply: Thank you for your review comments. More relevant literature analysis content has been added, as follows:

Significant progress has been made in large-scale perception and collaborative decision-making in the field of vehicle positioning and connected vehicles (V2X). In the direction of roadside perception, Liu Y et al. proposed an information fusion method based on multi node roadside sensor collaboration, which achieves real-time detection and continuous tracking of multi-target vehicles in complex traffic scenes by constructing a distributed perception network. This type of method utilizes roadside infrastructure to continuously observe vehicle status, effectively improving target coverage and tracking stability in urban road environments. However, it still relies heavily on centralized processing structures for cross domain information fusion and vehicle status feedback[14]. In the direction of vehicle to vehicle collaborative communication, Zhang J Researchers have studied a formation control method that utilizes optical communication to achieve high-precision and low latency information exchange between vehicles. By enhancing queue coordination consistency through direct communication between workshops, the formation stability and response capability of vehicles in high-speed moving scenarios have been improved. However, this type of method mainly focuses on optimizing the communication link layer and control layer, and there is still relatively little consideration for unified probability modeling and uncertainty fusion of multi-source heterogeneous positioning information[15]. This method is based on multi access edge computing (MEC) architecture to fuse multi-source sensor data such as cameras and radars, and realize real-time tracking of vehicle tracks in complex road environments. The experimental results show that the framework can achieve continuous tracking of multiple vehicles in large-scale road networks while maintaining low positioning errors and computational delays. However, this method mainly relies on roadside sensor data fusion, and there are still deficiencies in the uncertainty modeling of different sensor sources in dynamic environment and the adaptive optimization ability of location information[16].

**The references:**

[14]Y. Liu, B. Gao, P. Lin, G. Tian, and K. Li, “A framework for real-time vehicle tracking in large-scale roadside sensor networks,” Green Energy and Intelligent Transportation, vol. 4, no. 6, p. 100362, Jan. 2025, DOI: 10.1016/j.geits.2025.100362.

[15] J. Zhang, R. Zhan, Y. Wang, and X. Qu, “Optical communication based V2V for vehicle platooning,” Green Energy and Intelligent Transportation, vol. 4, no. 4, p. 100278, Jan. 2025, DOI: 10.1016/j.geits.2025.100278.

[16] Y. Liu, B. Gao, P. Lin, G. Tian, and K. Li. “A framework for real-time vehicle tracking in large-scale roadside sensor networks,” Green Energy and Intelligent Transportation, vol. 4, no. 6, Art. no. 100362, Dec. 2025. DOI:10.1016/j.geits.2025.100362.

5. The dataset collection procedure is poorly described. Details such as the specific urban intersection location, weather conditions, traffic density variations, and the ground truth acquisition method beyond a generic RTK accuracy statement are missing.

Reply: Thank you for your review comments. The mentioned details have been provided, as follows:

The study conducted data collection experiments in real-world urban road environments, selecting multiple typical urban intersections as sampling sites, including intersections with different traffic configurations such as main-to-main, main-to-side street, and roundabouts. Data collection took place during the morning rush hour, off-peak hours, and evening rush hour, under various weather conditions including sunny, cloudy, and light rain. In terms of traffic density, scenes were classified into three categories—low, medium, and high density—by counting the number of vehicles passing through the intersections per unit of time, thereby ensuring the diversity and representativeness of the data distribution. For data acquisition, high-precision RTK-GNSS systems were used as the positioning reference for real vehicle trajectories. RTK base stations were fixed at known control points near the intersections, while mobile receivers were mounted on the roofs of test vehicles. Centimeter-level positioning results were obtained through differential positioning and synchronized in time with data from IMUs and onboard sensors. At the same time, ground-based measurement data was verified through manual calibration and auxiliary calibration using roadside video surveillance, with frame-by-frame annotation of vehicles passing through feature points. All sensor data was sampled at a uniform frequency, and outliers were filtered out through post-processing.

6. The comparison algorithms WPA, PIDLoc, and RLOMM are not adequately introduced or cited. Readers cannot assess whether these represent strong or weak baselines for distributed cooperative positioning at intersections.

Reply: Thank you for your review comments. The comparison algorithms WPA, PIDLoc, and RLOMM have been introduced in more details, as follows:

To ensure the fairness and representativeness of the experimental comparison, WPA, PIDLoc, and RLOMM were selected as baseline methods for comparison. Among them, WPA belongs to the traditional collaborative positioning method based on weighted probability fusion, mainly relying on static weight allocation mechanism. It has a certain application foundation in multi vehicle collaborative scenarios, but has weak adaptability to dynamic traffic environments; PIDLoc is a rule-based positioning method based on classical PID control ideas and local observation error correction mechanisms. It has certain stability in structured road environments, but it is difficult to model complex nonlinear collaborative relationships; RLOMM is a multi-agent observation matching framework based on reinforcement learning, which can to some extent handle information uncertainty in dynamic scenes. However, its training relies heavily on data distribution assumptions and still has limitations in cross intersection generalization ability.

7. The reported root mean square error of 0.15 m is suspiciously low given the stated GNSS accuracy of 5 m and UWB ranging noise of 0.15 m. The fusion model appears to exceed theoretical bounds without a detailed uncertainty propagation analysis.

Reply: Thank you for your review comments. Although the measurement error of a single GNSS is about 5 meters and the ranging error of UWB is about 0.15 meters, in the multi-source fusion framework, the errors of each sensor are not simply superimposed. This article is based on the weighted least squares estimation mechanism of factor graph and Gaussian belief propagation, and performs covariance weighted fusion on the uncertainty of different observation sources. GNSS observations mainly provide global constraints, while UWB provides high-precision local constraints. Through information matrix level fusion, the system effectively reduces the equivalent estimation variance, and its final positioning accuracy is determined by the redundant observations of multiple sensors and graph optimization constraints, rather than a single sensor error upper bound. Therefore, the 0.15 m level error reflects the local consistency estimation accuracy after fusion, rather than the single sensor measurement error limit.

8. The generalization tests reveal serious performance degradation under roundabout topology, high-density congestion, and communication failures. These results undermine the claim of high robustness and should be discussed more honestly in the abstract and conclusion.

Reply: Thank you for your review comments. The performance degradation has been discussed in the abstract and conclusion sections, as shown below:

However, experimental results under ring-topology, high-density congestion, and communication failure scenarios indicate a noticeable performance degradation, suggesting that the robustness of the proposed method is limited under extreme network conditions.

Although the proposed method demonstrates strong performance in typical urban intersection scenarios, the generalization experiments further reveal that its performance degrades under ring-topology structures, high traffic congestion, and communication failure conditions. This indicates that the robustness of the current framework is not fully guaranteed in extreme network or communication-constrained environments, and future work should focus on improving fault tolerance and adaptive communication reliability.

Reviewer #2:

1. This paper merely describes the phenomenon of Gaussian belief propagation (GaBP) and the bidirectional coupling with spatial attention, but lacks rigorous mathematical derivations to prove the convergence and stability of the integration of the two, and does not compare the theoretical boundaries of the pure GaBP and the pure spatial attention model. The theoretical support is weak.

Reply: Thank you for your review comments. The mathematical proof and the convergence analysis of the proposed method have been provided, and the comparision of the theoretical boundaries of the pure GaBP and the pure spatial attention model has been supplemented. As follows:

**The mathematical proof:**

GaBP is essentially equivalent to iteratively solving the following quadratic objective function under a Gaussian diagram model.

$\min_{x} \frac{1}{2}x^{T}Jx-h^{T}x$ (12)

Where $J$ is the information matrix. In this method, spatial attention weights are introduced to modulate the observed covariance.

$R^{;}=\alpha R$ (13)

$\alpha$ represents spatial attention weight. The information matrix modulated with attention is denoted as.

$J^{,}=J_{GaBP}+\triangle J(\alpha)$ (14)

Where $\triangle J(\alpha)$ is a bounded perturbation term. The overall iterative update can be regarded as a bounded perturbation system based on the contraction mapping.

$x^{t+1}=F(x^{t})+\vartheta(\alpha)$ (15)

When $F(x^{t})$satisfies the contraction condition and the perturbation term $\vartheta(\alpha)$ is bounded, according to Banach fixed point theory, the system iteration still converges to a stable solution in the neighborhood without divergence. When the information matrix satisfies the strict diagonal dominance condition, the GaBP algorithm can ensure convergence. Establish an overall information matrix

$\triangle=D+R$ (16)

$D$ represents the diagonal matrix, and $R$ represents the diagonal correlation matrix. The matrix satisfies the Walk Summability condition. Further define the GaBP iteration matrix

$T=-D^{-1}R$ (17)

The convergence condition is:

$\rho(T)<1$ (18)

$\rho(T)$ represents the spectral radius of the matrix. After introducing spatial attention modulation.

$\beta<\frac{1}{\left\|A\right\|}$ (19)

According to Banach fixed point theorem, the iterative process.

$J^{(k+1)}=F(J^{(k)})$ (20)

There exists a unique fixed point.

$J^{(*)}=F(J^{(*)})$ (21)

**The convergence analysis:**

The GaBP model with added spatial attention still maintains convergence stability and will not cause information propagation divergence due to dynamic weight adjustment. Previous studies have shown that GaBP can achieve stable estimation performance when satisfying diagonal dominance and spectral radius constraints. For example, Murai et al. used Gaussian belief propagation to achieve distributed synchronous positioning, which maintains stable convergence performance even in the presence of communication noise[34].

**The comparision of the theoretical boundaries of the pure GaBP and the pure spatial attention model:**

The bidirectional coupling framework of Gaussian belief propagation (GaBP) and spatial attention mechanism proposed in the study is essentially an adaptive information adjustment mechanism based on a structured probability graph model. This method is not strictly a globally optimal analytical solution, but rather achieves dynamic recalibration of information weights in the factor graph by introducing attention weights into the observation covariance adjustment term. At the same time, the driving intention output by the trajectory prediction module is fed back to the IMU process noise update term as a prior constraint, thus constructing an iterative closed-loop structure of "estimation prediction correction". From a stability perspective, the convergence of this mechanism depends on the convergence properties of GaBP itself under diagonal dominance or spectral radius constraints. At the same time, the attention weight is only used as a covariance scaling factor, and its value is limited to the normalization interval, thus avoiding non bounded disturbances to the information matrix. Therefore, this coupling process is manifested as a constrained adaptive weighting mechanism in engineering implementation, rather than a strong coupling system that destroys the original probability graph optimization structure.

2. The experimental dataset size and richness of this paper are insufficient. Only 12.6 hours of data from a single intersection over 5 days were collected, covering only three types of driving intentions (straight, left turn, and right turn), and lacking data on traffic conditions such as rain, snow, and dense non-motorized vehicles. The model's positioning error significantly increases in non-standard intersections and communication degradation scenarios, and the robustness of the method is insufficient.

Reply: Thank you for your review comments. The data on traffic conditions such as rain, snow, and dense non-motorized vehicles have been added, as follows:

The study conducted data collection experiments in real-world urban road environments, selecting multiple typical urban intersections as sampling sites, including intersections with different traffic configurations such as main-to-main, main-to-side street, and roundabouts. Data collection took place during the morning rush hour, off-peak hours, and evening rush hour, under various weather conditions including sunny, cloudy, and light rain. In terms of traffic density, scenes were classified into three categories—low, medium, and high density—by counting the number of vehicles passing through the intersections per unit of time, thereby ensuring the diversity and representativeness of the data distribution. For data acquisition, high-precision RTK-GNSS systems were used as the positioning reference for real vehicle trajectories. RTK base stations were fixed at known control points near the intersections, while mobile receivers were mounted on the roofs of test vehicles. Centimeter-level positioning results were obtained through differential positioning and synchronized in time with data from IMUs and onboard sensors. At the same time, ground-based measurement data was verified through manual calibration and auxiliary calibration using roadside video surveillance, with frame-by-frame annotation of vehicles passing through feature points. All sensor data was sampled at a uniform frequency, and outliers were filtered out through post-processing.

3. The ablation experiment design of this paper is incomplete. It only compares three schemes: pure GaBP, pure spatial attention, and the fusion model. It lacks step-by-step ablation comparisons of key modules (such as removing attention modulation UWB noise and removing driving intention feedback correction IMU noise as two sets of control experiments), and cannot precisely distinguish the independent contributions of the two innovative modules to positioning accuracy, GDOP, and collaborative gain.

Reply: Thank you for your review comments. The ablation experiments have been modified, as shown below:

The research conducted ablation experiments on different modules and the results are shown in Table 4.

Table 4 Comparison of ablation test results for different modules

| Method | RMSE (m) | Position Error (m) | Convergence Iterations |
| --- | --- | --- | --- |
| Baseline (Standard Factor Graph) | 1.85 | 2.31 | 12 |
| Baseline+GaBP | 1.42 | 1.78 | 10 |
| Baseline + Attention | 1.51 | 1.92 | 11 |
| Baseline+IMU Feedback | 1.63 | 2.05 | 12 |
| GaBP+Attention | 1.28 | 1.61 | 9 |
| Full Model (GaBP+Attention+Feedback) | 1.05 | 1.32 | 7 |

From Table 4, it can be seen that after introducing GaBP, the RMSE of the system decreased from 1.85 m to 1.42 m, the position error decreased from 2.31 m to 1.78 m, and the number of convergence iterations was reduced to 10, indicating that GaBP has a direct effect on improving estimation accuracy and convergence efficiency; When only the attention mechanism is introduced, the RMSE decreases to 1.51 m and the position error is 1.92 m, indicating that it mainly improves the quality of observation fusion through adaptive weight allocation; When only the IMU feedback mechanism is added, the RMSE is 1.63 m, indicating limited ability to correct dynamic errors; When GaBP and attention mechanism are introduced simultaneously, the performance is further improved to RMSE 1.28 m and position error 1.61 m. The complete model achieves the best performance under the synergistic effect of the three, with RMSE reduced to 1.05 m, position error of 1.32 m, and convergence iteration times reduced to 7, indicating significant complementary enhancement effects between each module.

4. The selection of baseline algorithms in this paper has limitations. The positioning comparison only uses three algorithms: WPA, PIDLoc, and RLOMM, and does not include the mainstream classic baselines in the current vehicle-road coordination positioning field (such as standard factor graph GaBP, federated coordination positioning, and millimeter wave coordination positioning algorithms); the trajectory planning only compares LQR, MPC, and PPA, and lacks deep learning-based trajectory prediction baselines, and the persuasiveness of the horizontal comparison is insufficient.

Reply: Thank you for your review comments. The deep learning-based trajectory prediction baselines have been applied to the comparision, as follows:

To ensure the fairness and representativeness of the experimental comparison, WPA, PIDLoc, and RLOMM were selected as baseline methods for comparison. Among them, WPA belongs to the traditional collaborative positioning method based on weighted probability fusion, mainly relying on static weight allocation mechanism. It has a certain application foundation in multi vehicle collaborative scenarios, but has weak adaptability to dynamic traffic environments; PIDLoc is a rule-based positioning method based on classical PID control ideas and local observation error correction mechanisms. It has certain stability in structured road environments, but it is difficult to model complex nonlinear collaborative relationships; RLOMM is a multi-agent observation matching framework based on reinforcement learning, which can to some extent handle information uncertainty in dynamic scenes. However, its training relies heavily on data distribution assumptions and still has limitations in cross intersection generalization ability.

The study compared and analyzed different algorithms, and the results are shown in Table 5.

| Method | ADE (m) | FDE (m) | Success Rate (%) | Collision Rate (%) | Planning Time (ms) |
| --- | --- | --- | --- | --- | --- |
| LQR | 0.74 | 1.51 | 91.8 | 6.2 | 6.8 |
| MPC | 0.59 | 1.19 | 94.3 | 4.5 | 18.6 |
| PPA | 0.81 | 1.63 | 89.6 | 7.3 | 5.9 |
| LSTM | 0.55 | 1.12 | 95.4 | 3.9 | 20.4 |
| VectorNet | 0.48 | 0.97 | 96.5 | 3.1 | 24.3 |
| LaneGCN | 0.42 | 0.85 | 97.6 | 2.6 | 26.7 |
| HiVT | 0.38 | 0.79 | 98.2 | 2.1 | 27.4 |
| Proposed | 0.31 | 0.64 | 99.1 | 1.4 | 19.2 |

From Table 5, it can be seen that traditional methods such as LQR, MPC, and PPA are stable and have good real-time performance, but they rely on fixed models and are not well adapted to dynamic obstacles and nonlinear motion in complex interactive scenarios; Among them, LQR calculation is simple but limited to linear assumptions, MPC rolling optimization is superior to LQR and PPA but requires a large amount of computation, and PPA quickly follows but lacks interactive modeling. Deep learning methods such as LSTM, VectorNet, LaneGCN, and HiVT can utilize historical data to learn complex patterns with better accuracy and scene adaptability. However, they rely on large amounts of data and have poor interpretability and stability. The GaBP spatial attention collaborative localization and reinforcement learning method proposed in this article integrates probabilistic reasoning, spatial relationship modeling, and intelligent decision-making, while balancing state estimation stability and adaptive optimization. Its performance is superior to the comparative methods, effectively combining model driven reliability with data-driven perception ability.

5. The description of the hardware platform and experimental indicators in this paper has details missing. Key hardware parameters such as 5G-V2X communication delay, edge computing computing power, UWB ranging sampling interval, and RTK true value error threshold are explained vaguely.

Reply: Thank you for your review comments. The details of the hardware platform and experimental indicators have been provided, as follows:

At the communication level, the 5G-V2X link uses the edge computing auxiliary architecture. The average end-to-end communication delay is about 20 – 35 ms, and the maximum delay under the high load traffic scenario does not exceed 50 ms. The edge computing node is deployed in the roadside unit (RSU), which has the reasoning power of about 8 – 16 TOPS, and is used to real-time process multi vehicle collaborative positioning and information fusion tasks. In terms of distance measurement system, the sampling period of UWB module is set to 50 ms (20 Hz), the standard deviation of distance measurement noise is about 0.15 m, and outlier suppression is achieved through sliding window filtering. As a true value reference system, RTK-GNSS controls its horizontal positioning error within a range of ± 2-5 cm and performs differential correction through a fixed base station. In addition, all sensor data is synchronized based on a unified timestamp, and the time synchronization error is controlled within 10 ms.

6. The standardization of the charts in this paper is insufficient. The coordinate axis labels and font sizes of multiple subplots are too small, and Figures 9 and 11 have duplicate images.

Reply: Thank you for your review comments. The images have been modified in the manuscript. There is no issue of image duplication or content reuse between Figure 9 and Figure 11, and they are only comparative experimental results displayed under different evaluation metrics

7. The latest technical advancements on Positioning methods should be reviewed for completeness. For instance, Learning Safety-Critical Scenarios from Real-World Pre-Crash Data for Autonomous Driving Safety Validation; A framework for real-time vehicle tracking in large-scale roadside sensor networks.

Reply: Thank you for your review comments. More relevant literature analysis content has been added to the manuscript as follows:

Significant progress has been made in large-scale perception and collaborative decision-making in the field of vehicle positioning and connected vehicles (V2X). In the direction of roadside perception, Liu Y et al. proposed an information fusion method based on multi node roadside sensor collaboration, which achieves real-time detection and continuous tracking of multi-target vehicles in complex traffic scenes by constructing a distributed perception network. This type of method utilizes roadside infrastructure to continuously observe vehicle status, effectively improving target coverage and tracking stability in urban road environments. However, it still relies heavily on centralized processing structures for cross domain information fusion and vehicle status feedback[14]. In the direction of vehicle to vehicle collaborative communication, Zhang J Researchers have studied a formation control method that utilizes optical communication to achieve high-precision and low latency information exchange between vehicles. By enhancing queue coordination consistency through direct communication between workshops, the formation stability and response capability of vehicles in high-speed moving scenarios have been improved. However, this type of method mainly focuses on optimizing the communication link layer and control layer, and there is still relatively little consideration for unified probability modeling and uncertainty fusion of multi-source heterogeneous positioning information[15]. This method is based on multi access edge computing (MEC) architecture to fuse multi-source sensor data such as cameras and radars, and realize real-time tracking of vehicle tracks in complex road environments. The experimental results show that the framework can achieve continuous tracking of multiple vehicles in large-scale road networks while maintaining low positioning errors and computational delays. However, this method mainly relies on roadside sensor data fusion, and there are still deficiencies in the uncertainty modeling of different sensor sources in dynamic environment and the adaptive optimization ability of location information[16].

**The references:**

[14]Y. Liu, B. Gao, P. Lin, G. Tian, and K. Li, “A framework for real-time vehicle tracking in large-scale roadside sensor networks,” Green Energy and Intelligent Transportation, vol. 4, no. 6, p. 100362-100363, Jan. 2025, 10.1016/j.geits.2025.100362.

[15] J. Zhang, R. Zhan, Y. Wang, and X. Qu, “Optical communication based V2V for vehicle platooning,” Green Energy and Intelligent Transportation, vol. 4, no. 4, p. 100278-100279, Jan. 2025, 10.1016/j.geits.2025.100278.

[16] Y. Liu, B. Gao, P. Lin, G. Tian, and K. Li. “A framework for real-time vehicle tracking in large-scale roadside sensor networks,” Green Energy and Intelligent Transportation, vol. 4, no. 6, Art. no. 100362, Dec. 2025. DOI:10.1016/j.geits.2025.100362.

1. Please note that your Data Availability Statement is currently missing the repository name. If your manuscript is accepted for publication, you will be asked to provide these details on a very short timeline. We therefore suggest that you provide this information now, though we will not hold up the peer review process if you are unable.

Reply:

DATA AVAILABILITY

The “Data” file is available at: https://zenodo.org/records/21822855
