# Supplementary material for: Improving the accuracy of distributed cooperative positioning at urban intersections based on mathematical analysis and robust filtering
Source: PLoS One. 2026 Sep 24;21(9):e0358244. doi: 10.1371/journal.pone.0358244 (PMC13606937; doi:10.1371/journal.pone.0358244)
Supplement: Peer review history

# Peer review: Decision Letter 0

**DOI:** 10.1371/journal.pone.0358244.r001

**Authors:** Lei Zhang

21 Jun 2026

Dear Dr. Liu,

Thank you for submitting your manuscript to PLOS ONE. After careful consideration, we feel that it has merit but does not fully meet PLOS ONE’s publication criteria as it currently stands. Therefore, we invite you to submit a revised version of the manuscript that addresses the points raised during the review process.

Please submit your revised manuscript by Aug 20 2026 11:59PM. If you will need more time than this to complete your revisions, please reply to this message or contact the journal office at plosone@plos.org. When you're ready to submit your revision, log on to https://www.editorialmanager.com/pone/ and select the 'Submissions Needing Revision' folder to locate your manuscript file.

- A letter that responds to each point raised by the academic editor and reviewer(s). You should upload this letter as a separate file labeled 'Response to Reviewers'.
- A marked-up copy of your manuscript that highlights changes made to the original version. You should upload this as a separate file labeled 'Revised Manuscript with Track Changes'.
- An unmarked version of your revised paper without tracked changes. You should upload this as a separate file labeled 'Manuscript'.

If you would like to make changes to your financial disclosure, please include your updated statement in your cover letter. Guidelines for resubmitting your figure files are available below the reviewer comments at the end of this letter.

If applicable, we recommend that you deposit your laboratory protocols in protocols.io to enhance the reproducibility of your results. Protocols.io assigns your protocol its own identifier (DOI) so that it can be cited independently in the future. For instructions see: https://journals.plos.org/plosone/s/submission-guidelines#loc-laboratory-protocols. Additionally, PLOS ONE offers an option for publishing peer-reviewed Lab Protocol articles, which describe protocols hosted on protocols.io. Read more information on sharing protocols at https://plos.org/protocols?utm_medium=editorial-email&utm_source=authorletters&utm_campaign=protocols.

As the corresponding author, your ORCID iD is verified in the submission system and will appear in the published article. PLOS supports the use of ORCID, and we encourage all coauthors to register for an ORCID iD and use it as well. Please encourage your coauthors to verify their ORCID iD within the submission system before final acceptance, as unverified ORCID iDs will not appear in the published article. *Only*  the individual author can complete the verification step; PLOS staff *cannot*  verify ORCID iDs on behalf of authors.

We look forward to receiving your revised manuscript.

Kind regards,

Lei Zhang, PhD

Academic Editor

PLOS One

Journal Requirements:

When submitting your revision, we need you to address these additional requirements.

1. Please ensure that your manuscript meets PLOS ONE's style requirements, including those for file naming. The PLOS ONE style templates can be found at

https://journals.plos.org/plosone/s/file?id=wjVg/PLOSOne_formatting_sample_main_body.pdf and

https://journals.plos.org/plosone/s/file?id=ba62/PLOSOne_formatting_sample_title_authors_affiliations.pdf

2. Please note that PLOS One has specific guidelines on code sharing for submissions in which author-generated code underpins the findings in the manuscript. In these cases, we expect all author-generated code to be made available without restrictions upon publication of the work. Please review our guidelines at https://journals.plos.org/plosone/s/materials-and-software-sharing#loc-sharing-code and ensure that your code is shared in a way that follows best practice and facilitates reproducibility and reuse.

3. If the reviewer comments include a recommendation to cite specific previously published works, please review and evaluate these publications to determine whether they are relevant and should be cited. There is no requirement to cite these works unless the editor has indicated otherwise.

Additional Editor Comments:

Significant revisions are still needed in terms of technical clarity, experimental verification, writing coherence, etc.

[Note: HTML markup is below. Please do not edit.]

Reviewers' comments:

Reviewer's Responses to Questions

**Comments to the Author**

1. Is the manuscript technically sound, and do the data support the conclusions?

Reviewer #1: Yes

Reviewer #2: Yes

**********

2. Has the statistical analysis been performed appropriately and rigorously?

Reviewer #1: Yes

Reviewer #2: Yes

**********

3. Have the authors made all data underlying the findings in their manuscript fully available?

The PLOS Data policy

Reviewer #1: Yes

Reviewer #2: Yes

**********

4. Is the manuscript presented in an intelligible fashion and written in standard English?

Reviewer #1: Yes

Reviewer #2: Yes

**********

Reviewer #1: The paper presents a study on improving the accuracy of distributed cooperative positioning at urban intersections based on mathematical analysis and robust filtering. Based on the critical points below, I recommend major revision before the manuscript can be considered for publication.

1. The claimed theoretical innovation of bidirectionally coupling Gaussian belief propagation with spatial attention weights is not rigorously derived. No mathematical proof or convergence analysis is provided to demonstrate that the closed-loop feedback of driving intent into IMU noise covariance is stable or optimal.

2. The technical novelty is overstated. Integrating existing GaBP with a standard spatiotemporal attention network does not constitute a fundamentally new method, and similar factor graph-based collaborative positioning frameworks have been reported in prior literature.

3. The experimental validation lacks a proper ablation study. It is impossible to determine whether the performance gains come from the GaBP algorithm, the attention mechanism, or their coupling because no separate evaluation of each component under identical conditions is presented..

4. The latest advances on vehicle positioning and V2X should be reviewed for completeness. For instance, A framework for real-time vehicle tracking in large-scale roadside sensor networks; Optical communication based V2V for vehicle platooning.

5. The dataset collection procedure is poorly described. Details such as the specific urban intersection location, weather conditions, traffic density variations, and the ground truth acquisition method beyond a generic RTK accuracy statement are missing.

6. The comparison algorithms WPA, PIDLoc, and RLOMM are not adequately introduced or cited. Readers cannot assess whether these represent strong or weak baselines for distributed cooperative positioning at intersections.

7. The reported root mean square error of 0.15 m is suspiciously low given the stated GNSS accuracy of 5 m and UWB ranging noise of 0.15 m. The fusion model appears to exceed theoretical bounds without a detailed uncertainty propagation analysis.

8. The generalization tests reveal serious performance degradation under roundabout topology, high-density congestion, and communication failures. These results undermine the claim of high robustness and should be discussed more honestly in the abstract and conclusion.

Reviewer #2: 1. This paper merely describes the phenomenon of Gaussian belief propagation (GaBP) and the bidirectional coupling with spatial attention, but lacks rigorous mathematical derivations to prove the convergence and stability of the integration of the two, and does not compare the theoretical boundaries of the pure GaBP and the pure spatial attention model. The theoretical support is weak.

2. The experimental dataset size and richness of this paper are insufficient. Only 12.6 hours of data from a single intersection over 5 days were collected, covering only three types of driving intentions (straight, left turn, and right turn), and lacking data on traffic conditions such as rain, snow, and dense non-motorized vehicles. The model's positioning error significantly increases in non-standard intersections and communication degradation scenarios, and the robustness of the method is insufficient.

3. The ablation experiment design of this paper is incomplete. It only compares three schemes: pure GaBP, pure spatial attention, and the fusion model. It lacks step-by-step ablation comparisons of key modules (such as removing attention modulation UWB noise and removing driving intention feedback correction IMU noise as two sets of control experiments), and cannot precisely distinguish the independent contributions of the two innovative modules to positioning accuracy, GDOP, and collaborative gain.

4. The selection of baseline algorithms in this paper has limitations. The positioning comparison only uses three algorithms: WPA, PIDLoc, and RLOMM, and does not include the mainstream classic baselines in the current vehicle-road coordination positioning field (such as standard factor graph GaBP, federated coordination positioning, and millimeter wave coordination positioning algorithms); the trajectory planning only compares LQR, MPC, and PPA, and lacks deep learning-based trajectory prediction baselines, and the persuasiveness of the horizontal comparison is insufficient.

5. The description of the hardware platform and experimental indicators in this paper has details missing. Key hardware parameters such as 5G-V2X communication delay, edge computing computing power, UWB ranging sampling interval, and RTK true value error threshold are explained vaguely.

6. The standardization of the charts in this paper is insufficient. The coordinate axis labels and font sizes of multiple subplots are too small, and Figures 9 and 11 have duplicate images.

7. The latest technical advancements on Positioning methods should be reviewed for completeness. For instance, Learning Safety-Critical Scenarios from Real-World Pre-Crash Data for Autonomous Driving Safety Validation; A framework for real-time vehicle tracking in large-scale roadside sensor networks.

**********

what does this mean?). If published, this will include your full peer review and any attached files.

If you choose “no”, your identity will remain anonymous but your review may still be made public.

**Do you want your identity to be public for this peer review?**  For information about this choice, including consent withdrawal, please see our Privacy Policy

Reviewer #1: No

Reviewer #2: No

**********

[NOTE: If reviewer comments were submitted as an attachment file, they will be attached to this email and accessible via the submission site. Please log into your account, locate the manuscript record, and check for the action link "View Attachments". If this link does not appear, there are no attachment files.]

To ensure your figures meet our technical requirements, please review our figure guidelines: https://journals.plos.org/plosone/s/figures

You may also use PLOS’s free figure tool, NAAS, to help you prepare publication quality figures: https://journals.plos.org/plosone/s/figures#loc-tools-for-figure-preparation.

NAAS will assess whether your figures meet our technical requirements by comparing each figure against our figure specifications.

# Author comment: Author response to Decision Letter 1

**DOI:** 10.1371/journal.pone.0358244.r002

9 Aug 2026

The manuscript has been revised according to comments.

Attachment Submitted filename: Comments responsed 0807.doc

# Peer review: Decision Letter 1

**DOI:** 10.1371/journal.pone.0358244.r003

**Authors:** Lei Zhang

30 Aug 2026

Improving the Accuracy of Distributed Cooperative Positioning at Urban Intersections Based on Mathematical Analysis and Robust Filtering

PONE-D-26-27536R1

Dear Dr. Liu

We’re pleased to inform you that your manuscript has been judged scientifically suitable for publication and will be formally accepted for publication once it meets all outstanding technical requirements.

Within one week, you’ll receive an e-mail detailing the required amendments. When these have been addressed, you’ll receive a formal acceptance letter and your manuscript will be scheduled for publication.

An invoice will be generated when your article is formally accepted. Please note, if your institution has a publishing partnership with PLOS and your article meets the relevant criteria, all or part of your publication costs will be covered. Please make sure your user information is up-to-date by logging into Editorial Manager at Editorial Manager® and clicking the ‘Update My Information' link at the top of the page. For questions related to billing, please contact billing support.

If your institution or institutions have a press office, please notify them about your upcoming paper to help maximize its impact. If they’ll be preparing press materials, please inform our press team as soon as possible -- no later than 48 hours after receiving the formal acceptance. Your manuscript will remain under strict press embargo until 2 pm Eastern Time on the date of publication. For more information, please contact onepress@plos.org.

Kind regards,

Lei Zhang, PhD

Academic Editor

PLOS One

Additional Editor Comments (optional):

The revised paper is acceptable for publication.

Reviewers' comments:

Reviewer's Responses to Questions

**Comments to the Author**

Reviewer #1: All comments have been addressed

Reviewer #2: All comments have been addressed

**********

2. Is the manuscript technically sound, and do the data support the conclusions?

Reviewer #1: Yes

Reviewer #2: Yes

**********

3. Has the statistical analysis been performed appropriately and rigorously?

Reviewer #1: Yes

Reviewer #2: Yes

**********

4. Have the authors made all data underlying the findings in their manuscript fully available?

The PLOS Data policy

Reviewer #1: Yes

Reviewer #2: Yes

**********

5. Is the manuscript presented in an intelligible fashion and written in standard English?

Reviewer #1: Yes

Reviewer #2: Yes

**********

Reviewer #1: The revised paper has thoroughly resolved all my raised concerns. It can be accepted for publication.

Reviewer #2: (No Response)

**********

what does this mean?). If published, this will include your full peer review and any attached files.

If you choose “no”, your identity will remain anonymous but your review may still be made public.

**Do you want your identity to be public for this peer review?**  For information about this choice, including consent withdrawal, please see our Privacy Policy

Reviewer #1: No

Reviewer #2: No

**********

# Editor report: Acceptance letter

**DOI:** 10.1371/journal.pone.0358244.r004

**Authors:** Lei Zhang

PONE-D-26-27536R1

PLOS One

Dear Dr. Liu,

I'm pleased to inform you that your manuscript has been deemed suitable for publication in PLOS One. Congratulations! Your manuscript is now being handed over to our production team.

At this stage, our production department will prepare your paper for publication. This includes ensuring the following:

* All references, tables, and figures are properly cited

* All relevant supporting information is included in the manuscript submission,

* There are no issues that prevent the paper from being properly typeset

You will receive further instructions from the production team, including instructions on how to review your proof when it is ready. Please keep in mind that we are working through a large volume of accepted articles, so please give us a few days to review your paper and let you know the next and final steps.

Lastly, if your institution or institutions have a press office, please let them know about your upcoming paper now to help maximize its impact. If they'll be preparing press materials, please inform our press team within the next 48 hours. Your manuscript will remain under strict press embargo until 2 pm Eastern Time on the date of publication. For more information, please contact onepress@plos.org.

You will receive an invoice from PLOS for your publication fee after your manuscript has reached the completed accept phase. If you receive an email requesting payment before acceptance or for any other service, this may be a phishing scheme. Learn how to identify phishing emails and protect your accounts at https://explore.plos.org/phishing.

If we can help with anything else, please email us at customercare@plos.org.

Thank you for submitting your work to PLOS One and supporting open access.

Kind regards,

PLOS One Editorial Office Staff

on behalf of

Dr. Lei Zhang

Academic Editor

PLOS One
